# Cannabis use and suicide in people with a diagnosis of schizophrenia: a systematic review and meta-analysis of longitudinal, case control, and cross-sectional studies

**DOI:** 10.1017/S0033291725000236

**Published:** 2025-03-10

**Authors:** Lee D. Mulligan, Filippo Varese, Kamelia Harris, Gillian Haddock

**Affiliations:** 1 Division of Psychology and Mental Health, Faculty of Biology, Medicine and Health, School of Health Sciences, University of Manchester, Manchester Academic Health Science Centre (MAHSC), Manchester, UK; 2 Greater Manchester Mental Health NHS Foundation Trust (GMMH), Manchester Academic Health Science Centre (MAHSC), Manchester, UK; 3 Complex Trauma and Resilience Research Unit, Greater Manchester Mental Health NHS Foundation Trust (GMMH), Manchester, UK

**Keywords:** Cannabis, Suicide, Attempted Suicide, Suicidal Ideation, Schizophrenia, Meta-Analysis, Systematic Review

## Abstract

Cannabis use is highly prevalent in people with schizophrenia and is related to adverse clinical outcomes, including relapse and hospitalization. However, the relationship between cannabis and suicide remains inconclusive. This study aimed to systematically review and meta-analyze the relationship between cannabis use and suicide-related outcomes in people with schizophrenia. A comprehensive search of Medline, Embase, and PsycINFO for cross-sectional, case-control, and longitudinal studies was conducted using search terms from database inception to November 2024 inclusive. Computation of odds ratios (ORs) and hazard ratios (HRs) was performed using random effects models with DerSimonian-Laird estimation. All studies were appraised for quality. We also evaluated heterogeneity, publication bias and performed sub-group analyses and meta-regression. Twenty-nine studies comprising 36 samples met eligibility criteria. Cannabis use was not associated with odds of suicide death or suicidal ideation but was associated with risks of suicide death (HR = 1.21, 95% CI = 1.04 – 1.40) and odds of attempted suicide (OR = 1.40, 95% CI = 1.16 – 1.68). While between-sample heterogeneity was moderate in analyses of attempted suicide (*I*^2^ = 39.6%, *p* = 0.03), there was no publication bias. Summary effects remained significant in most sub-groups, but just failed to reach significance in longitudinal studies of attempted suicide (OR = 1.40, 95% CI = 0.97 – 1.68) and studies investigating first episode samples (OR = 1.24, 95% CI = 0.99 – 1.55). Cannabis use is significantly associated with some, but not all, suicide-related outcomes in people with schizophrenia. More work is needed to examine potential mechanisms of significant relationships.

## Introduction

Cannabis is one of the most used psychoactive drugs worldwide (Wang et al., 2024). Estimates suggest that each year approximately 219 million people use cannabis globally [United Nations Office on Drugs and Crime (UNODC), 2023], and this number is increasing (UNODC, 2013). The rise in cannabis use, coupled with its legalization and decriminalization in several high-income countries, has raised concerns about its potential impact upon mental health [World Health Organization (WHO), 2016]. Indeed, cannabis use has been associated with anxiety (Kedzior & Laeber, 2014), depression (Lev-Ran et al., 2014), and suicidality (Shamabadi et al., 2023) in clinical settings, and up to 22% of users meet criteria for cannabis use disorder (CUD) (Leung et al., 2020). Cannabis use has also been associated with severity of psychotic symptoms (Hindley et al., 2020) and a fourfold increased risk of psychosis (Marconi et al., 2016), a relationship that appears dose-dependent (Petrilli et al., 2022; Polkosnik et al., 2021).

Cannabis use is highly prevalent in people with a diagnosis of schizophrenia. Meta-analyses suggest the prevalence of CUD among this group is around 25% (Koskinen et al., 2010), and it is even higher (36%) in those with first episode psychosis (Hunt et al., 2018). Despite qualitative accounts of the beneficial effects of cannabis use on hopelessness (Asher & Gask, 2010), boredom, depression, anxiety, and psychotic symptoms (Parshotam & Joubert, 2015), and its restorative effect on identity and social networks (Wagstaff et al., 2018), cannabis use in schizophrenia has also been associated with deleterious clinical outcomes including relapse (Bioque et al., 2022), medication treatment resistance (Reid & Bhattacharyya, 2019), hospitalization (Patel et al., 2016), and suicide (Serafini et al., 2012).

Suicide is the leading cause of unnatural death among people with a diagnosis of schizophrenia (Moreno-Küstner et al., 2021). The risk of suicide is 10 times greater in this group compared to the general population (Correll et al., 2022), and the prevention of suicide is considered an international public health priority (Lu et al., 2020). Cannabis use is increasingly prevalent in those who die by suicide (Mulligan et al., 2024a) and has been associated with attempted suicide (Waterreus et al., 2018) and suicidal ideation (Ricci et al., 2023a) in people with a diagnosis of schizophrenia. This has led many to hypothesize a role of cannabinoid-1 receptor disruption (Ceccarini et al., 2015) and dysregulation of the endocannabinoid system more generally (Volkow, Hampson & Baler, 2017) in the emergence of suicidal behavior in this group. However, not all studies lend support for a link between cannabis and suicide (Heuschen et al., 2024; Naji et al., 2018; Reutfors et al., 2009). A small narrative review also concluded that recent data on this relationship is contradictory and insufficient to draw firm conclusions (Ricci et al., 2023b).

To the authors knowledge, no studies have attempted to meta-analyze all existing data on the relationship between cannabis use and suicide in people with a diagnosis of schizophrenia. This paper fills this important gap by synthesizing research on the relationship between cannabis use, measured categorically or continuously, of any frequency, degree, or impact, at any time point, and individual suicide-related outcomes (i.e., suicide death, attempted suicide, and suicidal ideation). By pooling and analyzing data from longitudinal, case control, and cross-sectional studies, we aimed to provide a quantitative assessment of the nature of these relationships while considering potential sources of heterogeneity and bias.

## Method

### Search strategy

A systematic review and meta-analysis, pre-registered with PROSPERO (registration number: CRD42024437152), were conducted following guidelines from the Preferred Reporting Items for Systematic Reviews and Meta-Analyses (PRISMA) (Moher et al., 2009). The process of identifying and retrieving eligible articles involved two strategies: (1) an electronic literature search across three major bibliographic databases; including Medline (spanning 1946 to 2024), PsycINFO (spanning 1806 to 2024), and Embase (spanning 1974 to 2024); and (2) examining the reference lists and citations of relevant papers to identify studies not found in the initial searches (i.e., forward and backward citation tracking).

The search incorporated terms related to substance use and suicide in its various manifestations, including (substance use* OR substance abuse* OR substance misuse* OR substance dependen* OR substanc* OR alcohol* OR alcohol use* OR alcohol abuse* OR alcohol misuse* OR alcohol dependen* OR addict* OR drug* OR drug use* OR drug abuse* OR drug misuse* OR drug dependence OR cannabis OR marijuana OR cocaine OR heroin OR amphetamine* OR methamphetamine* OR smoking OR tobacco OR nicotine).af AND (suicide OR suicid* OR suicidal behavio* OR self*harm*).af AND (psychosis OR psychoti* OR schizo* OR hallucinat* OR delusion* OR paranoi*).af. To broaden the search results and ensure all pertinent studies were identified, Medical Subject Headings (MeSH) were also utilized. Duplicate entries were systematically eliminated both electronically and manually. Broad search terms were used as a preliminary scoping review suggested that cannabis-related effects might be documented in studies addressing substance abuse in general, rather than exclusively focusing on cannabis.

### Inclusion and validity

The review included longitudinal, case-control, and cross-sectional studies that met the following criteria: (1) they involved adults diagnosed with a schizophrenia-spectrum disorder (such as psychotic disorder, schizophrenia, schizoaffective disorder, delusional disorder, or schizophreniform disorder) based on DSM-III, DSM-III-R, DSM-IV, DSM-IV-TR, DSM-V, or comparable research diagnostic criteria [such as the International Classification of Diseases (ICD)], or diagnoses confirmed through psychiatrist or psychologist case note reviews or clinical interviews; (2) they contained subjective or objective measures of cannabis use (including self-reported, informant-reported, or clinician-reported composite, dichotomous, or continuous measures of current or past cannabis use using questionnaires, checklists, or diagnoses from case note reviews, clinical interviews, or urine analyses); (3) they included a specific measure of suicidality (including self-reported, informant-reported, or clinician-reported dichotomous or continuous measures of suicide death, current or past suicide attempts or suicidal ideation); and (4) they provided summary estimates of cannabis use and suicide-related outcomes that were sufficient for meta-analysis.

We excluded studies that were not written in English or French. Additionally, we excluded (1) case reports, conference proceedings, and review articles; (2) studies focusing on non-suicidal self-injury (NSSI), given phenomenological differences between NSSI and suicidality in terms of intention, frequency, and lethality (Cipriano et al., 2017); (3) studies that used composite measures of suicidality or combined various suicide-related variables (e.g., grouping suicide with attempted suicide, or attempted suicide with suicidal ideation); and (4) studies examining adults with substance-induced psychosis, those ‘at risk’ of psychosis, focusing on retrospective relationships before the onset of psychosis, or those reporting on a mixed patient sample where less than 50% had a schizophrenia-spectrum diagnosis.

Two researchers independently assessed eligibility using a two-stage process: an initial screening of titles and abstracts, followed by a full-text review. During the initial phase, L.M. and K.H. independently screened all titles and abstracts. If either researcher found a title or abstract suitable for inclusion, it was progressed to full article screening (independent agreement: 92.5%). In the final phase, whole articles were reviewed to inform final decisions on inclusion (independent agreement: 98.5%). All discrepancies between the two researchers were resolved through consultation with a third reviewer (GH) to reach a consensus.

Risk of bias was evaluated using a modified version of the Effective Public Health Practice Project (EPHPP) tool (Thomas et al., 2004). This tool assessed the methodological quality of the studies across several domains, including (1) selection bias; (2) confounders; (3) data collection methods; (4) withdrawals/dropouts; and (5) statistical analyses, to provide an overall quality score. L.M. and K.H. independently assessed study quality and discrepancies were discussed and resolved with input from a third researcher (G.H.). There was substantial agreement on the global scores assigned by the coders (92.5%).

### Data extraction

All data were independently extracted by L.M. and K.H and were cross-checked to ensure accuracy. Authors were contacted if relevant data were missing from a study. Descriptive variables extracted included: year of study, country, study methodology, sample size, age of the sample, number and age of cases/controls with a suicide outcome (if applicable), gender, sample diagnoses, diagnosis measure, cannabis measure, cannabis use timeframe, suicidality measure, baseline total Positive and Negative Syndrome Scale (PANSS; Kay et al., 1987) score (if available), and statistical information needed to calculate effect sizes. The baseline total PANSS score was prioritized over other measures like the Brief Psychiatric Rating Scale (BPRS; Overall & Graham, 1988) as it includes both specific symptoms of schizophrenia and general psychopathology in one composite score.

To avoid data duplication, a hierarchy was constructed to guide decisions about extraction and improve the homogeneity of included studies. When studies reported multiple cannabis variables (e.g., binary and continuous), binary variables were preferentially chosen, as most studies measured cannabis by the presence or absence of use (or CUD). If cannabis use or specific suicide outcomes were recorded at various time points (e.g., recent and lifetime) or using self-reported and clinician reported measures, summary effects were pooled to create a single combined outcome metric. In cases where identical samples were used across different studies, eligible papers were selected sequentially based on pre-determined criteria: (1) the presence of binary cannabis variables, (2) the number of suicide outcome variables, or (3) sample size.

### Effect size computation and statistical analysis

All analyses were conducted using Stata version 14.0 (StataCorp, 2013). Summary effects were calculated using a random-effects model with DerSimonian-Laird estimation, anticipating variability in study methods, measures, and sample characteristics. Six models were developed to summarize effects across different suicide-related outcomes: (1) odds of suicide death, (2) risks of suicide death, (3) odds of attempted suicide, (4) risks of attempted suicide, (5) odds of suicidal ideation (including suicidal thoughts and plans), and (6) risks of suicidal ideation. The primary outcome metrics were Odds Ratios (ORs) and Hazard Ratios (HRs). When available, unadjusted ORs and HRs were extracted from all eligible studies. If these were not reported, adjusted HRs were used, or both ORs and their 95% confidence intervals were estimated from available descriptive statistics using 2 x 2 tables based on standard computational techniques for dichotomous data (Fleiss & Berlin, 2009). For studies presenting correlational or between-group analyses for continuous data, the correlation statistic, sample size, mean, standard deviation, and significance (*p*-value) were converted to ORs using Comprehensive Meta-Analysis (CMA) software (Borenstein, 2022).

Heterogeneity was assessed using Cochran’s Q and *I*^2^ statistics to determine and quantify the variance due to true heterogeneity (Higgins et al., 2003). An *I*^2^ value below 25% was considered low inconsistency, 25%–75% medium inconsistency, and above 75% high inconsistency. Publication and selection bias were evaluated by visual inspection of funnel plots and supplemented by Egger’s test for funnel plot asymmetry (Egger et al., 1997). Due to the risk of inflated type-1 error with Egger’s test, particularly for dichotomous outcomes with small sample sizes, it was only applied to outcomes with ten or more data points (Deeks et al., 2019). The ‘Trim-and-Fill’ method was also used to adjust for publication or selection bias on outcomes with ten or more data points (Duval & Tweedie, 2000).

Sensitivity analyses were performed to assess the impact of omitting specific studies on the overall summary estimates when studies held significant weighting or outliers were identified. For analyses with significant heterogeneity and including ten or more data points (Deeks et al., 2019), meta-regression analysis was used to explore whether factors such as publication year, age of sample, or baseline PANSS total score influenced the relationship between cannabis use and suicide-related outcomes. Subgroup analyses were also conducted to examine the effects of gender, study design, study quality, or illness course (first episode vs. chronic schizophrenia samples).

## Results

**Figure 1.**
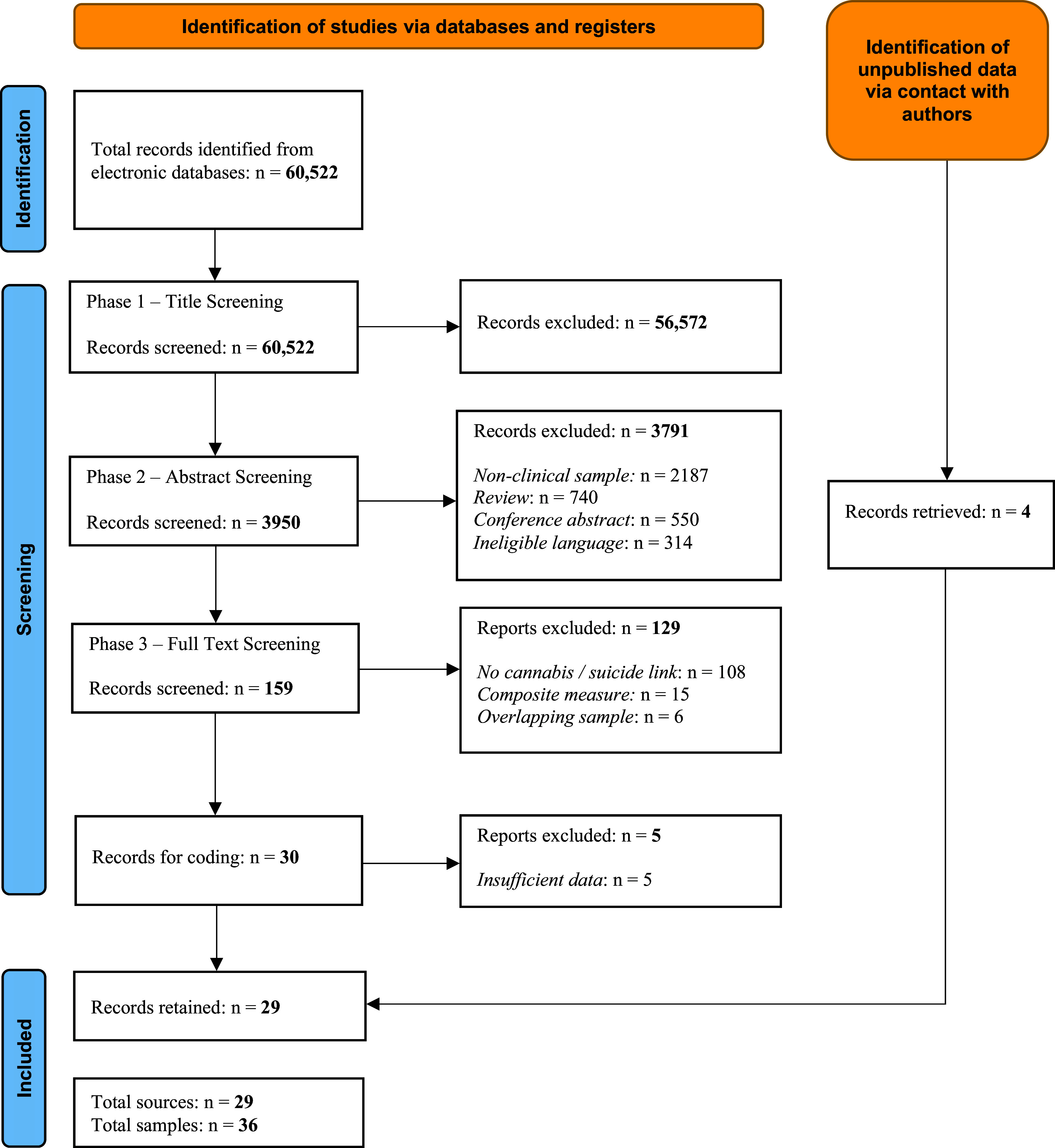
PRISMA Diagram.

60,522 studies were identified via database search. After title screening, 3,950 articles were screened by abstract, 159 full texts were scrutinized and 30 were included in the final coding phase. We excluded five studies at this stage as there was insufficient data to calculate summary effects. However, four of these studies were re-instated following contact with authors who shared previously unpublished data (Fekih-Romdhane et al., 2023; Fridman et al., 2023; Golay et al., 2023; Lahteenvuo et al., 2021). This resulted in 29 studies comprising 36 separate samples (see Figure 1). Of these, eight samples concerned cannabis and suicide death, 22 concerned cannabis and attempted suicide, and six concerned cannabis and suicidal ideation (See Table 1 and Appendix B and C).

**Table 1:.** Summary of eligible studies

Source	Study Methodology	Sample	Sample Size	No. of Cases (Suicide Outcome)	No. of Controls (No Suicide Outcome)
Suicide					
Reutfors et al, 2009 (Sweden)	Cross-Sectional / Case Control	Chronic	168	84	84
Dutta et al, 2011 (UK)	Longitudinal	FEP	1575	33	1542
Koola et al, 2012 (US)	Longitudinal	Chronic	762	3	759
Hjorthoj et al., 2015 (Denmark)	Longitudinal	Chronic	41,470	1138	40,332
Ostergaard et al., 2017 (Denmark)	Longitudinal	Chronic	35,625	1300	34,325
Lahteenvuo et al., 2021 (Finland)	Longitudinal	Chronic	30,860	8110	22,750
Lahteenvuo et al., 2021 (Sweden)	Longitudinal	Chronic	14,616	4514	10,102
Bornheimer et al, 2024 (USA)	Cross-Sectional / Case Control	Chronic	57	26	31
Attempted Suicide					
Dervaux et al, 2003 (France)	Cross-Sectional / Case Control	Chronic	34	18	16
Robinson et al, 2009 (Australia)	Cross-Sectional / Case Control	FEP	658	57	601
Makkos et al, 2011 (Hungary)	Cross-Sectional / Case Control	Chronic	85	17	68
McLean et al, 2012 (Australia)	Cross-Sectional / Case Control	Chronic	812	315	497
Mauri et al, 2013 (Italy)	Cross-Sectional / Case Control	Chronic	106	35	71
Luckhoff et al., 2014 (South Africa) – U, A,	Cross-Sectional / Case Control	Chronic	974	137	837
Ayesa-Arriola et al, 2015 (Spain)	Longitudinal	FEP	397	60	337
Adan et al, 2017 (Spain) – Males	Cross-Sectional / Case Control	Chronic	50	24	26
Ostergaard et al., 2017 (Denmark)	Longitudinal	Chronic	35,625	8763	26,862
Naji et al, 2018 (Canada) – Males	Cross-Sectional / Case Control	Chronic	364	-	-
Naji et al, 2018 (Canada) – Females	Cross-Sectional / Case Control	Chronic	314	-	-
Waterreus et al, 2018 (Australia) – Males	Cross-Sectional / Case Control	Chronic	1065	80	985
Waterreus et al, 2018 (Australia) – Females	Cross-Sectional / Case Control	Chronic	725	88	637
Lopez-Morinigo et al, 2019 (UK) – GAP	Cross-Sectional / Case Control	FEP	112	22	90
Toll et al, 2023 (Spain)	Cross-Sectional / Case Control	FEP	267	30	237
Fekih-Romdhane et al, 2023 (Tunisia)	Cross-Sectional / Case Control	FEP	33	5	28
Fridman et al, 2023 (Israel)	Cross-Sectional / Case Control	Chronic	144	130	14
Golay et al, 2023 (Switzerland)	Longitudinal	FEP	269	-	-
Koubaa et al, 2023 (Morocco)	Cross-Sectional / Case Control	Chronic	304	65	239
Sastre-Buades et al, 2023 (Spain)	Cross-Sectional / Case Control	FEP	190	30	160
Phalen et al, 2024 (USA)	Longitudinal	FEP	1101	-	-
Ricci et al, 2024 (Italy)	Longitudinal	FEP	42	22	20
Suicidal Ideation					
Salagre et al, 2020 (Spain)	Cross-Sectional / Case Control	FEP	302	23	279
Fridman et al, 2023 (Israel)	Cross-Sectional / Case Control	Chronic	144	112	32
Sicotte et al, 2023 (Canada)	Longitudinal	FEP	352	27	325
Heuschen et al, 2024 (The Netherlands)	Cross-Sectional / Case Control	FEP	551	-	-
Phalen et al, 2024 (USA)	Longitudinal	FEP	1101	-	-
Ricci et al, 2024 (Italy)	Longitudinal	FEP	42	22	20

*Note:* A, Abuse; D, Dependence; FEP, First Episode Psychosis; GAP, Genetics and Psychosis.

### Study characteristics

Twenty-nine studies were appraised: eighteen employed a cross-sectional/case control design and eleven utilized a longitudinal design. Studies took place at various sites worldwide: eighteen were conducted across Europe (Sweden, United Kingdom, Denmark, Finland, France, Hungary, Italy, Spain, Switzerland, and the Netherlands); four were conducted across North America (United States, Canada); three were conducted in Australia; three were conducted across Africa (Morocco, Tunisia, South Africa); and one was conducted in Asia (Israel). Overall, sixteen studies included people with a chronic schizophrenia diagnosis and thirteen utilized a first episode psychosis sample. Cannabis use was assessed primarily by the presence of absence of CUD: fifteen studies using ICD, DSM, DIGS, CIDI, or MINI diagnostic interviews; six via scrutiny of medical records; and four via study questionnaire. The remaining four studies utilized either validated measures comprising the Drug Use Scale (DUS; Drake et al., 1996), the Cannabis Use Disorder Identification Test – Revised (CUDIT-R; Adamson et al., 2010), and Cannabis Experience Questionnaire (CEQ_EU-GEI_; Di Forti et al., 2019) or clinician-rated items pertaining to the frequency of cannabis use over the last month.

### Quality assessment

Of the 29 eligible studies, one was deemed to be of weak quality, seventeen were deemed to be of moderate quality, and eleven were deemed to be of strong quality. Across studies, most common weaknesses were due to selection bias (representativeness of sample to wider population), and presence of confounders (See Appendix D).

### Association between cannabis use and suicide death

**Figure 2.**
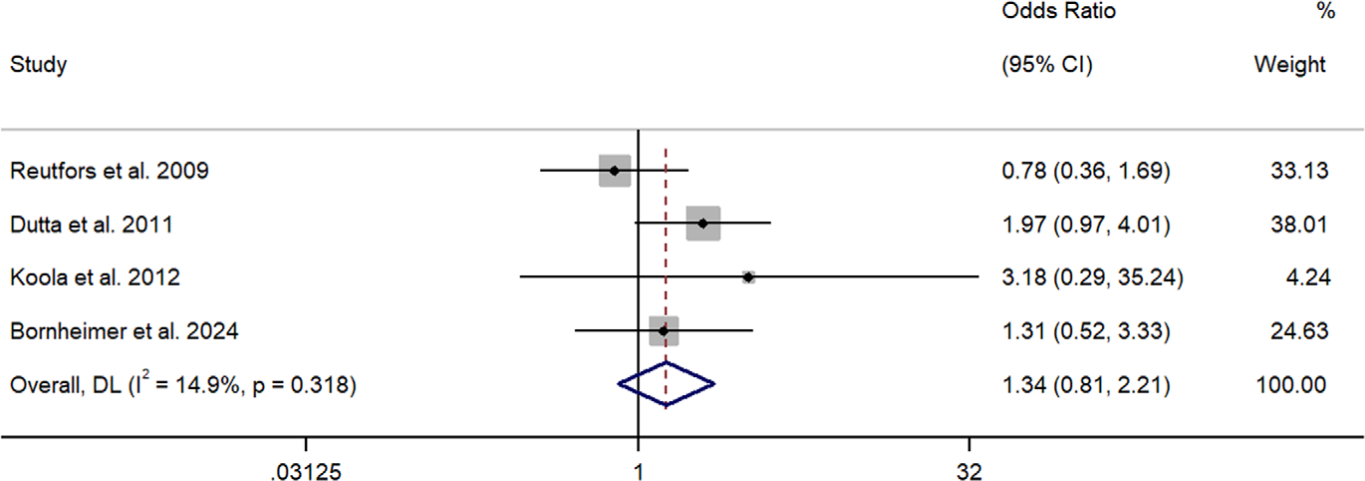
Forest plot for the meta-analysis examining odds ratios between cannabis use and suicide.

Four papers quantified the association between cannabis use and suicide death using ORs (Bornheimer et al., 2024; Dutta et al., 2011; Koola et al., 2012; Reutfors et al., 2009). Two studies employed a cross-sectional/case control design (Bornheimer et al., 2024; Reutfors et al., 2009), and two utilized a longitudinal design (Dutta et al., 2011; Koola et al., 2012). Utilizing a retrospective case control design, Reutfors et al. (2009) reported a non-significant relationship between cannabis use and suicide death in 84 age-matched cases from Sweden with a schizophrenia diagnosis. Bornheimer et al. (2024) reported a similar result utilizing a psychological autopsy method. They found that cannabis use was not significantly associated with suicide death in a mixed, heterogeneous, US sample of 57 patients. These studies were rated strong and moderate in quality, respectively, and suggest a non-significant relationship between cannabis use and suicide death in available cross-sectional studies. Comparably, a longitudinal, epidemiological study of several UK patient cohorts with FEP (*n* = 2,132) (i.e., London, Nottingham, Dumfries, and Galloway) found that cannabis use did not significantly predict suicide death over time (Dutta et al., 2011) nor did a US study of people with chronic schizophrenia (*n* = 762) (Koola et al., 2012). While these studies were rated as strong and moderate in quality, respectively, owing to few suicide cases in the latter study, this finding should be interpreted with caution. Collectively, the meta-analysis showed that cannabis use did not significantly increase the odds of suicide in people with schizophrenia (*k* = 4, OR = 1.34, 95% CI = 0.81 – 2.21) (See Figure 2). *Q* and *I*^2^ tests indicated that between-sample heterogeneity was low and non-significant (*I*^2^ = 14.9%, *p* = 0.32).

**Figure 3.**
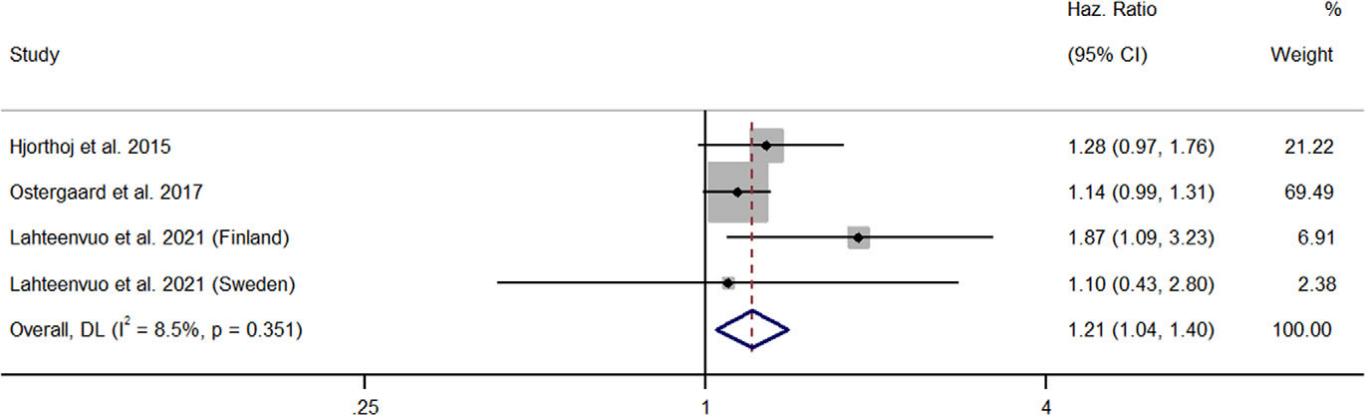
Forest plot for the meta-analysis examining hazard ratios between cannabis use and suicide.

Three papers comprising four samples reported HRs (Hjorthoj et al., 2015; Lahteenvuo et al., 2021; Ostergaard et al., 2017). All studies employed a longitudinal design and analyzed nation-wide, register-based cohorts, prospectively. In a large scale, cohort, study of Danish people with schizophrenia (*n* = 41,470), Hjorthoj et al. (2015) found that cannabis use was not significantly associated with risks of suicide death over time. However, this result just failed to reach significance. Ostergaard et al. (2017) reported similar findings in a separate cohort of Danish people with a schizophrenia-spectrum diagnosis (*n* = 35,625). Both current and past cannabis use were not significantly associated with risks of suicide, but only current use just failed to reach significance. Comparatively, whereas Lahteenvuo et al. (2021) found a significant relationship between cannabis use and suicide in a cohort of Finnish people with schizophrenia and substance use, this finding was not replicated in a parallel Swedish cohort. However, differences in the length of follow up could partially explain these results (Finnish cohort – 22 years, Swedish cohort – 11 years). All available longitudinal studies were rated moderate in quality and provided a mixed picture of the relationship between cannabis use and risks of suicide. Yet, collectively, the meta-analysis showed that cannabis use significantly increased the risk of suicide death in people with schizophrenia (*k* = 4, HR = 1.21, 95% CI = 1.04 – 1.40) (See Figure 3). Between-sample heterogeneity was low and non-significant (*I*^2^ = 8.5%, *p* = 0.35). This result persisted after the removal of studies holding significant weighting (Ostergaard et al., 2017) (*k* = 3, HR = 1.37, 95% CI = 1.07 – 1.77).

### Association between cannabis use and attempted suicide

**Figure 4.**
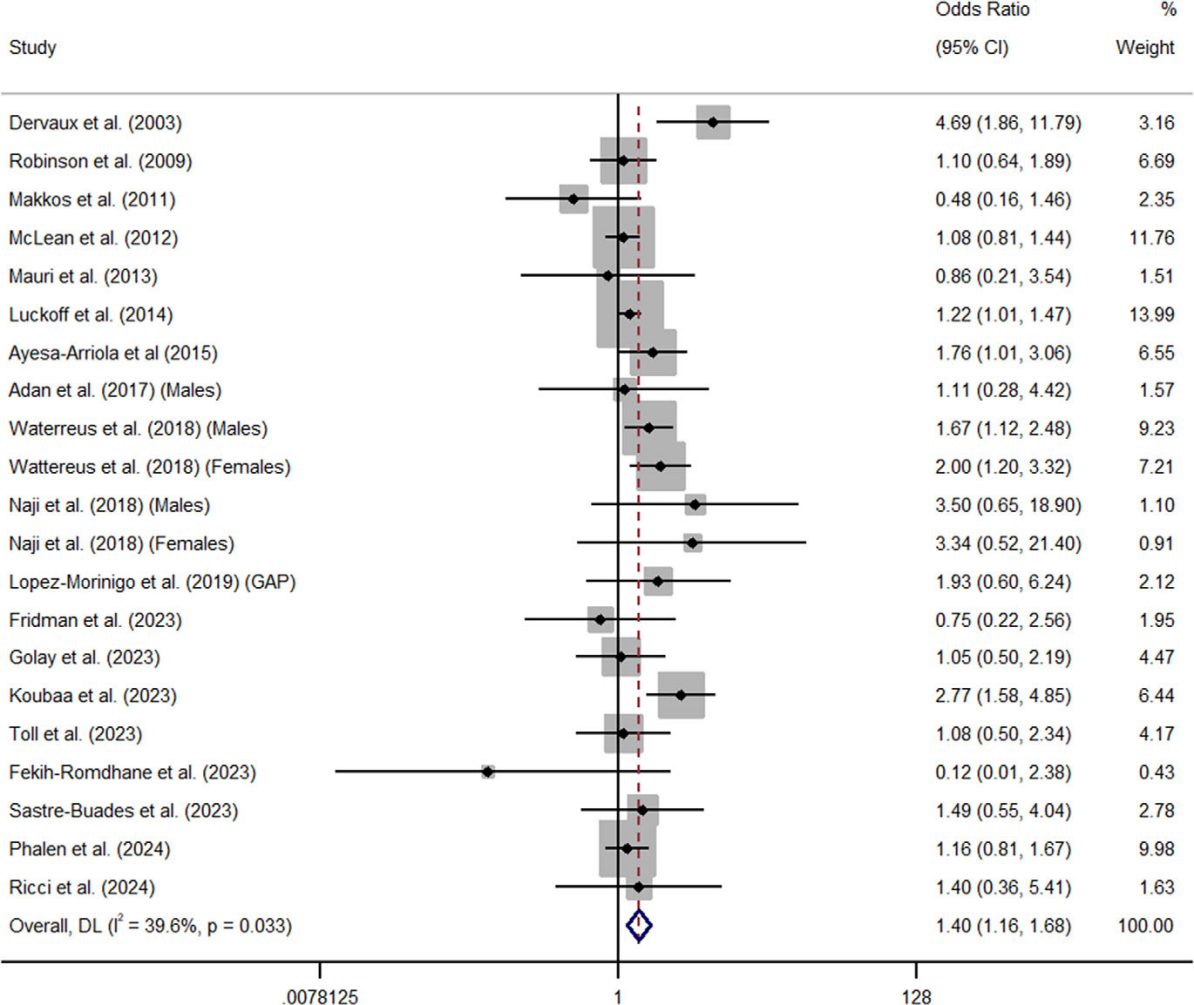
Forest plot for the meta-analysis examining odds ratios between cannabis use and attempted suicide.

Overall, cannabis use significantly increased the odds of attempted suicide in people with a diagnosis of schizophrenia (*k* = 21, OR = 1.40, 95% CI = 1.16 – 1.68) (See Figure 4). Furthermore, data from one large-scale, nation-wide, Danish prospective cohort study (n = 35,625) found that cannabis use significantly increased the risk of attempted suicide (HR = 1.16, 95% CI = 1.05 – 1.34) (Ostergaard et al., 2017). *Q* and *I*^2^ tests indicated that between-sample heterogeneity was medium and significant in analyses of odds between cannabis use and attempted suicide (*I*^2^ = 39.6%, *p* = 0.03). Publication or other selection bias was low and non-significant (*β* = .22, SE = .13, *p* = 0.55) (See Appendix E). Trim and Fill analysis also identified no missing studies. Across all analyses of attempted suicide, one study was an outlier. Sensitivity analyses revealed the relationship between cannabis use and attempted suicide decreased but remained significant following the removal of Dervaux et al. (2003) whose outcome metrics lay outside of the 95% CIs of the aggregated summary effect (*k* = 20, OR = 1.34, 95% CI = 1.14 – 1.57) (See Appendix F).

Subgroup analyses showed that cannabis use was significantly associated with attempted suicide in cross-sectional / case control studies (*k* = 17, OR = 1.44, 95% CI = 1.14 – 1.81, *I*^2^ = 48.7%, Q = 31.18), but effects just failed to reach significance in longitudinal studies (*k* = 4, OR = 1.40, 95% CI = 0.97 – 1.68, *I*^2^ = 0.0%, Q = 1.86) (See Appendix G & K). Furthermore, cannabis use was significantly associated with attempted suicide in studies using male only (*k* = 3, OR = 1.68, 95% CI = 1.16 – 2.44, *I*^2^ = 0%, Q = 1.08), female only (*k* = 2, OR = 2.07, 95% CI = 1.27 – 3.39, *I*^2^ = 0%, Q = 0.27), and gender combined samples (*k* = 14, OR = 1.33, 95% CI = 1.04 – 1.69, *I*^2^ = 49.9%, Q = 25.94) (See Appendix H & K). In addition, whereas summary effects of studies investigating samples with First Episode Psychosis (FEP) just failed to reach significance (*k* = 9, OR = 1.24, 95% CI = 0.99 – 1.55, *I*^2^ = 0.0%, Q = 5.70), those employing chronic presentations were significant (*k* = 12, OR = 1.53, 95% CI = 1.16 – 2.03, *I*^2^ = 59.2%, Q = 26.97) (See Appendix I & K). Summary effects were also significant in studies rated as strong (*k* = 9, OR = 1.25, 95% CI = 1.06 – 1,47, *I*^2^ = 0.0%, Q = 7.35) and moderate in methodological quality (*k* = 11, OR = 1.48, 95% CI = 1.01 – 2.17, *I*^2^ = 46.3%, Q = 18.61). One study rated weak in quality was also significant (Koubaa et al., 2023) (OR = 2.77, 95% CI = 1.58 – 4.85) (See Appendix J & K). Meta-regression showed that neither publication year (*k* = 21, *β* = 0.99, SE = 0.02, *p* = 0.79), mean age of cases (*k* = 9, *β* = 1.0, SE = 0.03, *p* = 0.86), nor baseline total PANSS score (*k* = 4, *β* = 0.99, SE = 0.04, *p* = 0.88) influenced the overall summary estimate.

### Association between cannabis use and suicidal ideation

Six papers provided statistical information sufficient to calculate the association between cannabis use and suicidal ideation using ORs (Fridman et al., 2023; Heuschen et al., 2024; Phalen et al., 2024; Ricci et al., 2024; Salagre et al., 2021; Sicotte et al., 2023). Two studies utilized a cross-sectional/case control design (Fridman et al., 2023; Heuschen et al., 2024) and four employed a longitudinal research design (Phalen et al., 2024; Ricci et al., 2024; Salagre et al., 2021; Sicotte et al., 2023).

Utilizing a retrospective cross-sectional design, Fridman et al. (2023) compared the frequency of suicidal ideation, extracted from medical records, in cannabis and non-cannabis using Israeli patients with a diagnosis of schizophrenia (*n* = 144). Reporting previously unpublished data, they found a non-significant relationship between cannabis use and suicidal ideation. In a larger study, Heuschen et al. (2024) reported similar findings in a Dutch cohort of individuals with FEP (*n* = 551). The regression found that cannabis use did not significantly predict suicidal ideation as measured by an item on the Community Assessment of Psychic Experiences (CAPE). Cross-sectional studies were categorized as moderate (Fridman et al., 2023) and strong (Heuschen et al., 2024) in quality and together suggest a non-significant relationship between cannabis use and suicidal ideation.

A similar pattern of non-significant results was found across all longitudinal studies. Ricci et al. (2024) compared differences in suicidal ideation between Italian patients with FEP (*n* = 57) grouped by self-reported cannabis, spice, and non-cannabis use. After combining results across multiple time-points, Ricci and colleagues found a non-significant relationship between cannabis use and suicidal ideation. Furthermore, employing similar research designs, both Salagre et al. (2021) and Sicotte et al. (2023) compared cannabis use over time in groups categorised by trajectory of suicidal ideation (i.e., non vs. worsening, and low vs. persistent, respectively). Both studies found non-significant relationships between cannabis use and suicidal ideation in both Canadian (*n* = 382) and Spanish (*n* = 334) samples with FEP. Lastly, in a study of US patients with FEP (*n* = 1101), Phalen et al. (2024) reported relationships between cannabis use and both clinician-rated and self-reported suicidal ideation. When combined, they found a non-significant relationship between cannabis use and suicidal ideation, though this just failed to reach significance. All longitudinal studies were rated as moderate in quality, except for Phalen colleagues (2024), which was rated as strong.

**Figure 5.**
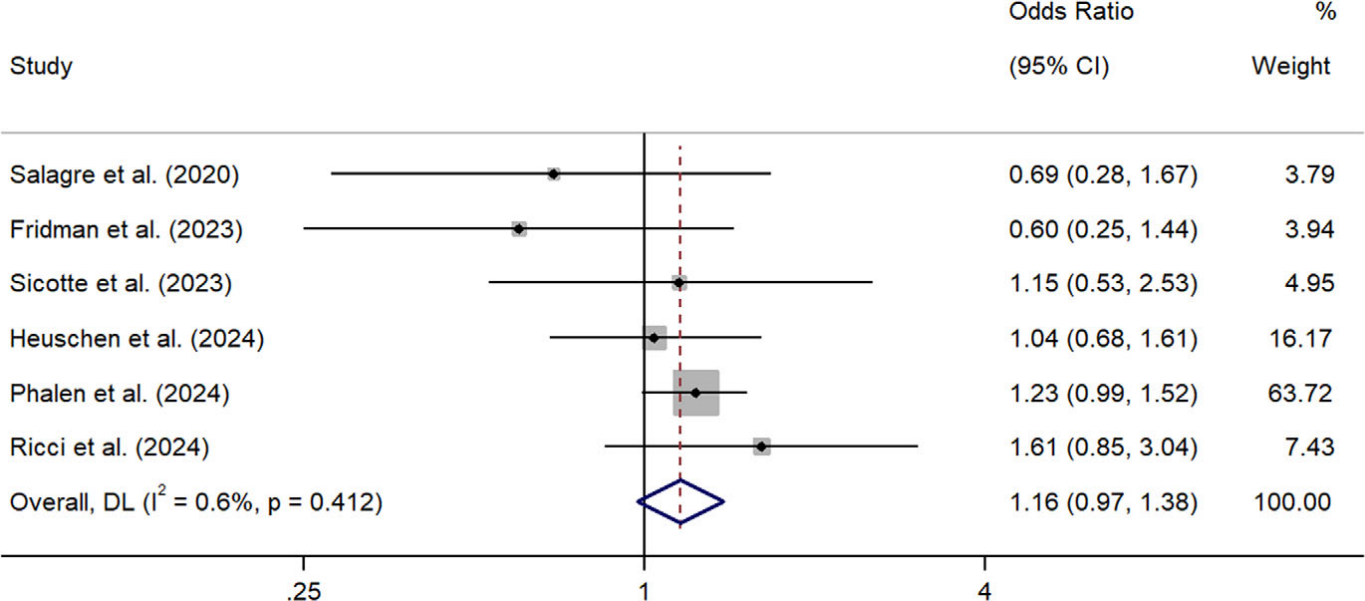
Forest plot for the meta-analysis examining odds ratios between cannabis use and suicidal ideation.

Collectively, the meta-analysis showed that cannabis did not significantly increase the odds of suicidal ideation in people with a diagnosis of schizophrenia, but this just failed to reach significance (*k* = 6, OR = 1.16, 95% CI = 0.97 – 1.38) (See Figure 5). Between sample heterogeneity was low and non-significant (*I*^2^ = 0.6%, *p* = 0.41). The summary effect was further reduced after the removal of studies holding significant weighting (Phalen et al., 2024) (*k* = 5, OR = 1.04, 95% CI = 0.77 – 1.40).

## Discussion

This review found that cannabis use is significantly associated with some, but not all, suicide-related outcomes in people with a diagnosis of schizophrenia. Specifically, while cannabis use was not associated with odds of suicide death or suicidal ideation, it was associated with risks of death and odds of attempted suicide. Summary effects remained significant in most sub-groups (i.e., in cross-sectional studies, in studies rated as strong, moderate or weak in quality, in studies comprising male-only, female-only, and gender combined samples and in studies sampling individuals with chronic schizophrenia), but just failed to reach significance in longitudinal studies of attempted suicide and studies investigating attempted suicide in first episode samples. While there was evidence of moderate homogeneity, there was no evidence of publication bias and no missing studies. Neither publication year, sample age or PANSS score significantly predicted the summary estimates.

Our findings advance existing work on the relationship between cannabis use and suicide-related outcomes in people with a diagnosis of schizophrenia. Existing reviews have concluded that research supporting the link between cannabis and suicide in this group is inconclusive (Ricci et al., 2023b; Serafini et al., 2012). However, by grouping studies by suicide outcome, we found evidence for a significant link between cannabis use, risks of suicide, and odds of attempted suicide specifically. These findings lend support for individual studies reporting positive associations between cannabis use and both suicide death and attempted suicide (Koubaa et al., 2023; Lahteenvuo et al., 2021; Waterreus et al., 2018) and suggest that cannabis use should be considered a risk factor for adverse outcomes (WHO, 2016). Interestingly, we did not find a significant link between cannabis use and either odds of suicide death or suicidal ideation, especially following the removal of studies holding significant weighting. This lends partial support for anecdotal qualitative accounts on the potential buffering effects of cannabis use on hopelessness, boredom, and anxiety (Asher & Gask, 2010; Parshotam & Joubert, 2015), experiences known to precipitate suicidal thinking (Ben-Zeev et al., 2012; Chong et al., 2020). Furthermore, the discrepancy in significance between cannabis use and suicide death as measured by reporting of ORs or HRs could suggest that while cannabis might not confer a significantly greater odds of suicide death, it might influence the speed at which suicide deaths occur in people with a diagnosis of schizophrenia.

Several potential mechanisms could underlie the summary effects observed in this meta-analysis. Firstly, cannabis, and specifically THC, could directly affect serotonin and neurotransmitter function to produce symptoms of depression and / or other mental health conditions known to increase suicidal risk and attempts (Pedersen, 2008). Moreover, the role of cannabinoid-1 receptor disruption and dysregulation of the endocannabinoid system following cannabis use have also been implicated in the emergence of suicidal behavior (Ceccarini et al., 2015; Volkow et al., 2017). Secondly, cannabis use might be indirectly related to suicide via other mechanisms. It is widely known that cannabis intoxication produces changes in behavioral, cognitive, and motor functions, including impaired problem-solving and impulsivity (Trull et al., 2016). Cannabis use has also been associated with antipsychotic medication treatment resistance (Reid & Bhattacharyya, 2019), non-compliance (Patel et al., 2020), and symptomatic relapse (Bioque et al., 2022; Hindley et al., 2020), all of which correlate with suicide attempts (Huang et al., 2018). However, future studies employing repeated, longitudinal measures of psychosis, medication adherence, cognition, impulsivity, cannabis use, and suicidality are required to examine temporal relationships between these variables and permit more nuanced conclusions regarding underlying mediators and moderators. Lastly, it could be that cannabis use is related to suicide and attempted suicide in combination with other substances. Epidemiological data suggests cannabis might be a gateway drug that enhances vulnerability to the rewarding effects of other substances (Anthony, 2012), including alcohol (Ceccarini et al., 2014), itself correlated with suicidal outcomes (Mulligan et al., 2024b). Therefore, future research should attempt to disentangle the relative effects of individual substances on suicide-related outcomes to inform a greater understanding of drug-specific mechanisms.

To our knowledge, this is the first ever attempt to quantify the relationship between cannabis use and suicide-related outcomes in people with a diagnosis of schizophrenia and explore potential sources of heterogeneity. We also included unpublished data from existing sources (Fekih-Romdhane et al., 2023; Fridman et al., 2023; Golay et al., 2023; Lahteenvuo et al., 2021). However, our findings should be considered in the context of the following limitations. Firstly, the summary effects for suicide death and suicidal ideation, and sub-group analyses for attempted suicide, were based on a small number of pooled studies. Whilst our searches were exhaustive, the small number of eligible studies means some reported summary effects may lack precision and should therefore be interpreted cautiously. Secondly, a binary measure of cannabis use (i.e., absence or presence of CUD) was chosen as the main predictor across all analyses as this was reported by most primary studies. The amount of THC could not be determined or examined. This is notable as there is evidence that the onset and frequency of cannabis use as well as cannabis potency may be more important predictors of adverse outcomes, including suicide, in people with psychosis (Di Forti et al., 2014; Quattrone et al., 2021). There are also differences in the route of cannabis exposure (i.e., inhalation vs. ingestion) and clinical outcome (Monte et al., 2019), which could explain between study differences in reported summary effects. Finally, it is unclear whether or how the legality of THC cannabis may lead to biases in data collection and participant selection. Therefore, the representativeness of the samples included in this meta-analysis could be questionable.

In conclusion, cannabis use is significantly associated with some, but not all, suicide-related outcomes in people with a diagnosis of schizophrenia. Specifically, summary effects were significant for suicide risks and attempted suicide, which persisted across most sub-groups. However, there was no significant relationship with suicide odds or suicidal ideation. While further research is needed to examine potential mechanisms of significant effects, clinicians should inquire about cannabis use, inform patients and carers about its effects, and support those who wish to abstain as part of routine clinical practice, psychoeducation and suicide-risk prevention. This is important given the increasing prevalence and continued legalization and de-criminalization of cannabis use globally (UNODC, 2023, 2013; Wang et al., 2024) and international public health priorities regarding the prevention of suicide in people with a diagnosis of schizophrenia.

## Supporting information

Mulligan et al. supplementary material Mulligan et al. supplementary material

## Supplementary material

The supplementary material for this article can be found at http://doi.org/10.1017/S0033291725000236.

## Data Sharing

Please contact the corresponding author if you would like to see any data that are not included in the Article or the appendix.

## References

[r1] Adamson, S. J., Kay-Lambkin, F. J., Baker, A. L., Lewin, T. J., Thornton, L., Kelly, B. J., & Sellman, J. D. (2010). An improved brief measure of cannabis misuse: the Cannabis Use Disorders Identification Test-Revised (CUDIT-R). Drug and Alcohol Dependence, 110(1-2), 137–143. 20347232 10.1016/j.drugalcdep.2010.02.017

[r2] Adan, A., Capella, M. D. M., Prat, G., Forero, D. A., López-Vera, S., & Navarro, J. F. (2017). Executive functioning in men with schizophrenia and substance use disorders Influence of lifetime suicide attempts. PloS One, 12(1), e0169943. 28099526 10.1371/journal.pone.0169943 PMC5242526

[r3] Anthony, J. C. (2012). Steppingstone and gateway ideas: a discussion of origins, research challenges, and promising lines of research for the future. Drug and Alcohol Dependence, 123, S99–S104. 22572210 10.1016/j.drugalcdep.2012.04.006 PMC4900966

[r4] Asher, C. J., & Gask, L. (2010). Reasons for illicit drug use in people with schizophrenia: Qualitative study. BMC Psychiatry, 10, 1–15. 21092168 10.1186/1471-244X-10-94 PMC2999587

[r5] Ayesa-Arriola, R., Alcaraz, E. G., Hernández, B. V., Pérez-Iglesias, R., Moríñigo, J. D. L., Duta, R., & Crespo-Facorro, B. (2015). Suicidal behaviour in first-episode non-affective psychosis: Specific risk periods and stage-related factors. European Neuropsychopharmacology, 25(12), 2278–2288. 26475577 10.1016/j.euroneuro.2015.09.008

[r6] Ben-Zeev, D., Young, M. A., & Depp, C. A. (2012). Real-time predictors of suicidal ideation: mobile assessment of hospitalized depressed patients. Psychiatry Research, 197(1-2), 55–59. 22397912 10.1016/j.psychres.2011.11.025

[r7] Bioque, M., Mezquida, G., Amoretti, S., García-Rizo, C., López-Ilundain, J. M., Diaz-Caneja, C. M., & Bernardo, M. (2022). Clinical and treatment predictors of relapse during a three-year follow-up of a cohort of first episodes of schizophrenia. Schizophrenia Research, 243, 32–42. 35231832 10.1016/j.schres.2022.02.026

[r8] Borenstein, M. (2022). Comprehensive meta-analysis software. In M. Egger, J. P. T. Higgins, & G. D. Smith (Eds.), Systematic Reviews in Health Research: Meta-analysis in Context (3rd ed., pp. 535–548). John Wiley & Sons.

[r9] Bornheimer, L. A., Bagge, C., Overholser, J., Brdar, N. M., Matta, N., Kitchen, M., & Stockmeier, C. A. (2024). Demographic and clinical characteristics of individuals with psychosis symptoms who died by suicide: Findings of a psychological autopsy study. Psychiatry Research, 116185. 39288536 10.1016/j.psychres.2024.116185

[r10] Ceccarini, J., Hompes, T., Verhaeghen, A., Casteels, C., Peuskens, H., Bormans, G., & Van Laere, K. (2014). Changes in cerebral CB1 receptor availability after acute and chronic alcohol abuse and monitored abstinence. Journal of Neuroscience, 34(8), 2822–2831. 24553924 10.1523/JNEUROSCI.0849-13.2014 PMC6608522

[r11] Ceccarini, J., Kuepper, R., Kemels, D., van Os, J., Henquet, C., & Van Laere, K. (2015). [18 F] MK-9470 PET measurement of cannabinoid CB 1 receptor availability in chronic cannabis users. Addiction Biology, 20(2), 357–367. 24373053 10.1111/adb.12116

[r13] Chong, B. T. W., Wahab, S., Muthukrishnan, A., Tan, K. L., Ch’ng, M. L., & Yoong, M. T. (2020). Prevalence and factors associated with suicidal ideation in institutionalized patients with schizophrenia. Psychology Research & Behavior Management, 949–962. 33204188 10.2147/PRBM.S266976 PMC7667143

[r14] Cipriano, A., Cella, S., & Cotrufo, P. (2017). Nonsuicidal self-injury: A systematic review. Frontiers in Psychology, 8, 1946. 29167651 10.3389/fpsyg.2017.01946 PMC5682335

[r15] Correll, C. U., Solmi, M., Croatto, G., Schneider, L. K., Rohani-Montez, S. C., Fairley, L., & Tiihonen, J. (2022). Mortality in people with schizophrenia: a systematic review and meta-analysis of relative risk and aggravating or attenuating factors. World Psychiatry, 21(2), 248–271. 35524619 10.1002/wps.20994 PMC9077617

[r16] Deeks, J. J., Higgins, J. P., & Altman, D. G., & Cochrane Statistical Methods Group. (2019). Analysing data and undertaking meta-analyses. In J. P. T. Higgins, J. Thomas, J. Chandler, M. Cumpston, T. Li, M. J. Page, & V. A. Welch (Eds.), Cochrane Handbook for Systematic Reviews of Interventions (2nd ed., pp. 241–284). John Wiley & Sons.

[r17] Dervaux, A., Laqueille, X., Bourdel, M.-C., LeBorgne, M.-H., Olié, J.-P., Lóo, H., & Krebs, M.-O. (2003). Cannabis et schizophrénie: Données cliniques et socio-démographiques [Cannabis and schizophrenia: Demographic and clinical correlates]. L’Encéphale: Revue de psychiatrie clinique biologique et thérapeutique, 29(1), 11–17. 12640322

[r18] Di Forti, M., Sallis, H., Allegri, F., Trotta, A., Ferraro, L., Stilo, S. A., & Murray, R. M. (2014). Daily use, especially of high-potency cannabis, drives the earlier onset of psychosis in cannabis users. Schizophrenia Bulletin, 40(6), 1509–1517. 24345517 10.1093/schbul/sbt181 PMC4193693

[r19] Di Forti, M., Quattrone, D., Freeman, T. P., Tripoli, G., Gayer-Anderson, C., Quigley, H., & van der Ven, E. (2019). The contribution of cannabis use to variation in the incidence of psychotic disorder across Europe (EU-GEI): a multicentre case-control study. The Lancet Psychiatry, 6(5), 427–436. 30902669 10.1016/S2215-0366(19)30048-3 PMC7646282

[r20] Drake, R. E., Mueser, K. T., & McHugo, G. J. (1996). Clinician rating scales: alcohol use scale (AUS), drug use scale (DUS), and substance abuse treatment scale (SATS). Outcomes Assessment in Clinical Practice, 113(6), 2.

[r21] Dutta, R., Murray, R. M., Allardyce, J., Jones, P. B., & Boydell, J. (2011). Early risk factors for suicide in an epidemiological first episode psychosis cohort. Schizophrenia Research, 126(1-3), 11–19. 21183318 10.1016/j.schres.2010.11.021

[r22] Duval, S., & Tweedie, R. (2000). Trim and fill: A simple funnel-plot–based method of testing and adjusting for publication bias in meta-analysis. Biometrics, 56(2), 455–463. 10877304 10.1111/j.0006-341x.2000.00455.x

[r23] Egger, M., Smith, G. D., Schneider, M., & Minder, C. (1997). Bias in meta-analysis detected by a simple, graphical test. British Medical Journal, 315(7109), 629–634. 9310563 10.1136/bmj.315.7109.629 PMC2127453

[r24] Fekih-Romdhane, F., Abassi, B., Ghrissi, F., Loch, A. A., Cherif, W., Damak, R., & Cheour, M. (2023). Suicide risk among individuals at Ultra-High Risk (UHR) of psychosis in a developing North African country: a 12-month naturalistic prospective cohort study from the TRIP project. Psychiatry Research, 327, 115409. 37633155 10.1016/j.psychres.2023.115409

[r25] Fleiss, J. L., & Berlin, J. A. (2009). Effect sizes for dichotomous data. The Handbook of Research Synthesis and Meta-Analysis, 2, 237–253.

[r26] Fridman, J., Bloemhof-Bris, E., Weizman, S., Kessler, T., Porat, D., Ivry, A., & Shelef, A. (2023). Inflammation Markers Among Schizophrenia Patients Who Use Cannabis. Clinical Neuropharmacology, 46(4), 145–148. 10.1097/WNF.0000000000000558 37335845

[r27] Golay, P., Reitzel, E., & Conus, P. (2023). The impact of established risk factors for psychosis on the 3-year outcomes. Swiss Archives of Neurology, Psychiatry & Psychotherapy, 174(03), 88–92.

[r28] Heuschen, C. B. B. C. M., Bolhuis, K., Zantvoord, J. B., Bockting, C. L., Denys, D. A. J. P., Lok, A., … & Schirmbeck, F. (2024). Self-reported suicidal ideation among individuals with first episode psychosis and healthy controls: Findings from the international multicentre EU-GEI study. Schizophrenia Research, 270, 339–348. 38968805 10.1016/j.schres.2024.06.041

[r29] Higgins, J. P., Thompson, S. G., Deeks, J. J., & Altman, D. G. (2003). Measuring inconsistency in meta-analyses. British Medical Journal, 327(7414), 557–560. 12958120 10.1136/bmj.327.7414.557 PMC192859

[r30] Hindley, G., Beck, K., Borgan, F., Ginestet, C. E., McCutcheon, R., Kleinloog, D., … & Howes, O. D. (2020). Psychiatric symptoms caused by cannabis constituents: a systematic review and meta-analysis. The Lancet Psychiatry, 7(4), 344–353. 32197092 10.1016/S2215-0366(20)30074-2 PMC7738353

[r31] Hjorthøj, C., Østergaard, M. L. D., Benros, M. E., Toftdahl, N. G., Erlangsen, A., Andersen, J. T., & Nordentoft, M. (2015). Association between alcohol and substance use disorders and all-cause and cause-specific mortality in schizophrenia, bipolar disorder, and unipolar depression: a nationwide, prospective, register-based study. The Lancet Psychiatry, 2(9), 801–808. 26277044 10.1016/S2215-0366(15)00207-2

[r33] Huang, X., Fox, K. R., Ribeiro, J. D., & Franklin, J. C. (2018). Psychosis as a risk factor for suicidal thoughts and behaviors: a meta-analysis of longitudinal studies. Psychological Medicine, 48(5), 765–776. 28805179 10.1017/S0033291717002136

[r34] Hunt, G. E., Large, M. M., Cleary, M., Lai, H. M. X., & Saunders, J. B. (2018). Prevalence of comorbid substance use in schizophrenia spectrum disorders in community and clinical settings, 1990–2017: Systematic review and meta-analysis. Drug & Alcohol Dependence, 191, 234–258. 30153606 10.1016/j.drugalcdep.2018.07.011

[r35] Kay, S. R., Fiszbein, A., & Opler, L. A. (1987). The positive and negative syndrome scale (PANSS) for schizophrenia. Schizophrenia Bulletin, 13(2), 261–276. 3616518 10.1093/schbul/13.2.261

[r36] Kedzior, K. K., & Laeber, L. T. (2014). A positive association between anxiety disorders and cannabis use or cannabis use disorders in the general population-a meta-analysis of 31 studies. BMC Psychiatry, 14, 1–22. 10.1186/1471-244X-14-136 PMC4032500 24884989

[r37] Koola, M. M., McMahon, R. P., Wehring, H. J., Liu, F., Mackowick, K. M., Warren, K. R., & Kelly, D. L. (2012). Alcohol and cannabis use and mortality in people with schizophrenia and related psychotic disorders. Journal of Psychiatric Research, 46(8), 987–993. 22595870 10.1016/j.jpsychires.2012.04.019 PMC3392453

[r38] Koskinen, J., Löhönen, J., Koponen, H., Isohanni, M., & Miettunen, J. (2010). Rate of cannabis use disorders in clinical samples of patients with schizophrenia: a meta-analysis. Schizophrenia Bulletin, 36(6), 1115–1130. 19386576 10.1093/schbul/sbp031 PMC2963055

[r39] Koubaa, I., Aden, M. O., & Barrimi, M. (2023). Prevalence and factors associated with suicide attempts among Moroccan patients with schizophrenia: cross-sectional study. Annals of Medicine & Surgery, 85(6), 2528–2533. 37363523 10.1097/MS9.0000000000000771 PMC10289528

[r40] Lähteenvuo, M., Batalla, A., Luykx, J. J., Mittendorfer-Rutz, E., Tanskanen, A., Tiihonen, J., & Taipale, H. (2021). Morbidity and mortality in schizophrenia with comorbid substance use disorders. Acta Psychiatrica Scandinavica, 144(1), 42–49. 33650123 10.1111/acps.13291 PMC8359349

[r41] Leung, J., Chan, G. C., Hides, L., & Hall, W. D. (2020). What is the prevalence and risk of cannabis use disorders among people who use cannabis? A systematic review and meta-analysis. Addictive Behaviors, 109, 106479. 32485547 10.1016/j.addbeh.2020.106479

[r42] Lev-Ran, S., Roerecke, M., Le Foll, B., George, T. P., McKenzie, K., & Rehm, J. (2014). The association between cannabis use and depression: a systematic review and meta-analysis of longitudinal studies. Psychological Medicine, 44(4), 797–810. 23795762 10.1017/S0033291713001438

[r43] Lopez-Morinigo, J. D., Di Forti, M., Ajnakina, O., Wiffen, B. D., Morgan, K., Doody, G. A., & David, A. S. (2019). Insight and risk of suicidal behaviour in two first-episode psychosis cohorts: Effects of previous suicide attempts and depression. Schizophrenia Research, 204, 80–89. 30253893 10.1016/j.schres.2018.09.016

[r44] Lu, L., Dong, M., Zhang, L., Zhu, X. M., Ungvari, G. S., Ng, C. H., & Xiang, Y. T. (2020). Prevalence of suicide attempts in individuals with schizophrenia: a meta-analysis of observational studies. Epidemiology & Psychiatric Sciences, 29, e39. 10.1017/S2045796019000313 PMC8061230 31172899

[r45] Lückhoff, M., Koen, L., Jordaan, E., & Niehaus, D. (2014). Attempted suicide in a Xhosa schizophrenia and schizoaffective disorder population. Suicide & Life-Threatening Behavior, 44(2), 167–174. 24286498 10.1111/sltb.12066

[r46] Makkos, Z., Fejes, L., Inczédy-Farkas, G., Kassai-Farkas, A., Faludi, G., & Lazary, J. (2011). Psychopharmacological comparison of schizophrenia spectrum disorder with and without cannabis dependency. Progress in Neuro-Psychopharmacology & Biological Psychiatry, 35(1), 212–217. 21087649 10.1016/j.pnpbp.2010.11.007

[r47] Marconi, A., Di Forti, M., Lewis, C. M., Murray, R. M., & Vassos, E. (2016). Meta-analysis of the association between the level of cannabis use and risk of psychosis. Schizophrenia Bulletin, 42(5), 1262–1269. 26884547 10.1093/schbul/sbw003 PMC4988731

[r48] Mauri, M. C., Paletta, S., Maffini, M., Moliterno, D., & Altamura, A. C. (2013). Suicide attempts in schizophrenic patients: clinical variables. Asian Journal of Psychiatry, 6(5), 421–427. 24011691 10.1016/j.ajp.2013.07.001

[r49] McLean, D., Gladman, B., & Mowry, B. (2012). Significant relationship between lifetime alcohol use disorders and suicide attempts in an Australian schizophrenia sample. Australian & New Zealand Journal of Psychiatry, 46(2), 132–140. 22311529 10.1177/0004867411433211

[r50] Moher, D., Liberati, A., Tetzlaff, J., Altman, D. G., & PRISMA Group*, T. (2009). Preferred reporting items for systematic reviews and meta-analyses: the PRISMA statement. Annals of Internal Medicine, 151(4), 264–269. 19622511 10.7326/0003-4819-151-4-200908180-00135

[r51] Monte, A. A., Shelton, S. K., Mills, E., Saben, J., Hopkinson, A., Sonn, B., & Abbott, D. (2019). Acute illness associated with cannabis use, by route of exposure: an observational study. Annals of Internal Medicine, 170(8), 531–537. 30909297 10.7326/M18-2809 PMC6788289

[r52] Moreno-Küstner, B., Guzman-Parra, J., Pardo, Y., Sanchidrián, Y., Díaz-Ruiz, S., & Mayoral-Cleries, F. (2021). Excess mortality in patients with schizophrenia spectrum disorders in Malaga (Spain): A cohort study. Epidemiology & Psychiatric Sciences, 30, e11. 33536113 10.1017/S2045796020001146 PMC8057505

[r53] Mulligan, L. D., Bojanić, L., Hunt, I. M., Baird, A., Turnbull, P., Kapur, N., & Shaw, J. (2024a). Substance use and self-poisoning in schizophrenia: 11-year findings from a national clinical survey of suicide in mental health patients in the UK. Schizophrenia Research, 267, 254–260. 38581828 10.1016/j.schres.2024.03.048

[r54] Mulligan, L. D., Varese, F., Harris, K., & Haddock, G. (2024b). Alcohol use and suicide-related outcomes in people with a diagnosis of schizophrenia: a comprehensive systematic review and meta-analysis. Psychological Medicine, 54(1), 1–12. 37818642 10.1017/S0033291723002738

[r55] Naji, L., Rosic, T., Dennis, B., Bhatt, M., Sanger, N., Hudson, J., & Samaan, Z. (2018). The association between cannabis use and suicidal behavior in patients with psychiatric disorders: an analysis of sex differences. Biology of Sex Differences, 9, 1–8. 29891008 10.1186/s13293-018-0182-x PMC5996511

[r56] Østergaard, M. L., Nordentoft, M., & Hjorthøj, C. (2017). Associations between substance use disorders and suicide or suicide attempts in people with mental illness: a Danish nation-wide, prospective, register-based study of patients diagnosed with schizophrenia, bipolar disorder, unipolar depression or personality disorder. Addiction, 112(7), 1250–1259. 28192643 10.1111/add.13788

[r57] Overall, J. E., & Gorham, D. R. (1988). The Brief Psychiatric Rating Scale (BPRS): Recent developments in ascertainment and scaling. Psychopharmacology Bulletin, 24(1), 97–99. 3387516

[r58] Parshotam, R. K., & Joubert, P. M. (2015). Views of schizophrenia patients on the effects of cannabis on their mental health. South African Journal of Psychiatry, 21(2), 57–61.

[r59] Patel, R., Wilson, R., Jackson, R., Ball, M., Shetty, H., Broadbent, M., & Bhattacharyya, S. (2016). Association of cannabis use with hospital admission and antipsychotic treatment failure in first episode psychosis: an observational study. BMJ Open, 6(3), e009888. 10.1136/bmjopen-2015-009888 PMC4785290 26940105

[r60] Patel, R. S., Sreeram, V., Vadukapuram, R., & Baweja, R. (2020). Do cannabis use disorders increase medication non-compliance in schizophrenia? United States Nationwide inpatient cross-sectional study. Schizophrenia Research, 224, 40–44. 33183946 10.1016/j.schres.2020.11.002

[r61] Pedersen, W. (2008). Does cannabis use lead to depression and suicidal behaviours? A population-based longitudinal study. Acta Psychiatrica Scandinavica, 118(5), 395–403. 18798834 10.1111/j.1600-0447.2008.01259.x

[r62] Petrilli, K., Ofori, S., Hines, L., Taylor, G., Adams, S., & Freeman, T. P. (2022). Association of cannabis potency with mental ill health and addiction: a systematic review. The Lancet Psychiatry, 9(9), 736–750. 35901795 10.1016/S2215-0366(22)00161-4

[r63] Phalen, P., Jones, N., Davis, B., Sarpal, D., Dickerson, F., Vatza, C., & Bennett, M. (2024). Suicidality among clients in a network of coordinated specialty care (CSC) programs for first-episode psychosis: Rates, changes in rates, and their predictors. Schizophrenia Research, 274, 150–157. 39298811 10.1016/j.schres.2024.07.054 PMC12118571

[r64] Polkosnik, G. L., Sorkhou, M., & George, T. P. (2021). Effects of cannabis use on psychotic and mood symptoms: a systematic review. Canadian Journal of Addiction, 12(3), 10–21.

[r65] Quattrone, D., Ferraro, L., Tripoli, G., La Cascia, C., Quigley, H., Quattrone, A., & Di Forti, M. (2021). Daily use of high-potency cannabis is associated with more positive symptoms in first-episode psychosis patients: the EU-GEI case–control study. Psychological Medicine, 51(8), 1329–1337. 32183927 10.1017/S0033291720000082 PMC8223239

[r66] Reid, S., & Bhattacharyya, S. (2019). Antipsychotic treatment failure in patients with psychosis and co-morbid cannabis use: A systematic review. Psychiatry Research, 280, 112523. 31450032 10.1016/j.psychres.2019.112523

[r67] Reutfors, J., Brandt, L., Jönsson, E. G., Ekbom, A., Sparén, P., & Ösby, U. (2009). Risk factors for suicide in schizophrenia: findings from a Swedish population-based case-control study. Schizophrenia Research, 108(1-3), 231–237. 19176276 10.1016/j.schres.2008.12.023

[r68] Ricci, V., Ceci, F., Di Carlo, F., Di Muzio, I., Ciavoni, L., Santangelo, M., & Maina, G. (2023a). First episode psychosis with and without the use of cannabis and synthetic cannabinoids: Psychopathology, global functioning and suicidal ideation. Psychiatry Research, 320, 115053. 36682093 10.1016/j.psychres.2023.115053

[r69] Ricci, V., Cristofori, E., Passarello, E., Paggi, A., Cavallo, A., Ceci, F., & Maina, G. (2023b). Cannabis use and suicide in non-affective psychosis: a mini-review of recent literature. Psychiatria Danubina, 35(3), 307–319. 37917836 10.24869/psyd.2023.307

[r70] Ricci, V., Di Muzio, I., Ceci, F., Di Carlo, F., Mancusi, G., Piro, T., & Maina, G. (2024). Aberrant salience in cannabis-induced psychosis: a comparative study. Frontiers in Psychiatry, 14, 1343884. 38260781 10.3389/fpsyt.2023.1343884 PMC10801803

[r71] Robinson, J., Cotton, S., Conus, P., Graf Schimmelmann, B., McGorry, P., & Lambert, M. (2009). Prevalence and predictors of suicide attempt in an incidence cohort of 661 young people with first-episode psychosis. Australian & New Zealand Journal of Psychiatry, 43(2), 149–157. 19153923 10.1080/00048670802607162

[r72] Salagre, E., Grande, I., Jiménez, E., Mezquida, G., Cuesta, M. J., Llorente, C., & PEPs Group. (2021). Trajectories of suicidal ideation after first-episode psychosis: A growth mixture modeling approach. Acta Psychiatrica Scandinavica, 143(5), 418–433. 33501646 10.1111/acps.13279

[r73] Sastre-Buades, A., Caro-Cañizares, I., Ochoa, S., Lorente-Rovira, E., Barajas, A., Gutiérrez-Zotes, A., & Spanish Metacognition Study Group. (2023). Relationship between cognition and suicidal behavior in recent-onset psychosis. Schizophrenia Research, 252, 172–180. 36652834 10.1016/j.schres.2022.12.042

[r74] Serafini, G., Pompili, M., Innamorati, M., Rihmer, Z., Sher, L., & Girardi, P. (2012). Can cannabis increase the suicide risk in psychosis? A critical review. Current Pharmaceutical Design, 18(32), 5165–5187. 22716157 10.2174/138161212802884663

[r75] Shamabadi, A., Ahmadzade, A., Pirahesh, K., Hasanzadeh, A., & Asadigandomani, H. (2023). Suicidality risk after using cannabis and cannabinoids: An umbrella review. Dialogues in Clinical Neuroscience, 25(1), 50–63. 37427882 10.1080/19585969.2023.2231466 PMC10334849

[r76] Sicotte, R., Iyer, S. N., Lacourse, É., Séguin, J. R., & Abdel-Baki, A. (2023). Heterogeneity in the course of suicidal ideation and its relation to suicide attempts in first-episode psychosis: a 5-year prospective study. The Canadian Journal of Psychiatry, 68(11), 850–859. 37071553 10.1177/07067437231167387 PMC10590090

[r77] StataCorp, L. P. (2013). Stata multilevel mixed-effects reference manual. College Station, TX: StataCorp.

[r78] Thomas, B. H., Ciliska, D., Dobbins, M., & Micucci, S. (2004). A process for systematically reviewing the literature: Providing the research evidence for public health nursing interventions. Worldviews on Evidence-Based Nursing, 1(3), 176–184. 17163895 10.1111/j.1524-475X.2004.04006.x

[r79] Toll, A., Pechuan, E., Bergé, D., Legido, T., Martínez-Sadurní, L., El-Abidi, K., … & Mané, A. (2023). Factors associated with suicide attempts in first-episode psychosis during the first two years after onset. Psychiatry Research, 325, 115232. 37146463 10.1016/j.psychres.2023.115232

[r80] Trull, T. J., Wycoff, A. M., Lane, S. P., Carpenter, R. W., & Brown, W. C. (2016). Cannabis and alcohol use, affect and impulsivity in psychiatric out-patients’ daily lives. Addiction, 111(11), 2052–2059. 27270874 10.1111/add.13471 PMC5056804

[r81] United Nations Office on Drugs and Crome (UNODC). (2023). World Drug Report 2023. United Nations. https://www.unodc.org/res/WDR-2023/WDR23_Exsum_fin_DP.pdf

[r82] United Nations Office on Drugs and Crome (UNODC). (2013). World Drug Report 2013. United Nations. https://www.unodc.org/unodc/secured/wdr/wdr2013/World_Drug_Report_2013.pdf

[r83] Volkow, N. D., Hampson, A. J., & Baler, R. D. (2017). Don’t worry, be happy: endocannabinoids and cannabis at the intersection of stress and reward. Annual Review of Pharmacology & Toxicology, 57(1), 285–308. 10.1146/annurev-pharmtox-010716-104615 27618739

[r84] Wagstaff, C., Graham, H., Farrell, D., Larkin, M., & Tatham, L. (2018). Perspectives of cannabis use in the life experience of men with schizophrenia. International Journal of Mental Health Nursing, 27(3), 1099–1108. 29218823 10.1111/inm.12422

[r85] Wang, Q., Qin, Z., Xing, X., Zhu, H., & Jia, Z. (2024). Prevalence of Cannabis Use around the World: A Systematic Review and Meta-Analysis, 2000–2024. China CDC Weekly, 6(25), 597–604. 38933041 10.46234/ccdcw2024.116 PMC11196877

[r86] Waterreus, A., Di Prinzio, P., Badcock, J. C., Martin-Iverson, M., Jablensky, A., & Morgan, V. A. (2018). Is cannabis a risk factor for suicide attempts in men and women with psychotic illness? Psychopharmacology, 235, 2275–2285. 29766209 10.1007/s00213-018-4924-6

[r87] World Health Organisation (WHO). (2016). The health and social effects of nonmedical cannabis use. World Health Organisation. https://iris.who.int/bitstream/handle/10665/251056/9789241510240-eng.pdf?sequence=1

