# Supplementary material for: Cannabis use and suicide in people with a diagnosis of schizophrenia: a systematic review and meta-analysis of longitudinal, case control, and cross-sectional studies
Source: Psychol Med. 2025 Mar 10;55:e79. doi: 10.1017/S0033291725000236 (PMC12080644; doi:10.1017/S0033291725000236)
Supplement: Mulligan et al. supplementary material [file S0033291725000236sup001.docx]

SUPPLEMENTARY APPENDIX

**Cannabis use and suicide in people with a diagnosis of schizophrenia: a systematic review and meta-analysis of longitudinal, case control and cross-sectional studies.**

*Dr. Lee D. Mulligan, ClinPsyD

Prof. Filippo Varese, PhD

Dr. Kamelia Harris, PhD

Prof. Gillian Haddock, PhD

*Corresponding author: Dr. Lee Mulligan, Division of Psychology and Mental Health, University of Manchester, Manchester, UK ([lee.mulligan@manchester.ac.uk](mailto:lee.mulligan@manchester.ac.uk)).

**Contents**

Supplementary Appendix A: PRISMA Checklist

Supplementary Appendix B: Demographic information of included studies (i)

Supplementary Appendix C: Demographic information of included studies (ii)

Supplementary Appendix D: Risk of Bias Assessment – Cannabis / Suicide-Related Outcomes

Supplementary Appendix E: Publication Bias - Cannabis / Attempted Suicide (k = 21) (ORs)

Supplementary Appendix F: Sensitivity Analysis - Cannabis / Attempted Suicide (ORs)

Supplementary Appendix G: Subgroup Analysis by Research Design – Cannabis / Attempted Suicide (ORs)

Supplementary Appendix H: Subgroup Analysis by Gender – Cannabis / Attempted Suicide (ORs)

Supplementary Appendix I: Subgroup Analysis by Illness Course – Cannabis / Attempted Suicide (ORs)

Supplementary Appendix J: Subgroup Analysis by Quality – Cannabis / Attempted Suicide (ORs)

Supplementary Appendix K: Subgroup Analysis Table – Cannabis / Attempted Suicide (ORs)

Supplementary References

**Supplementary Appendix A**: PRISMA Checklist

| **Section and Topic** | **Item #** | **Checklist item** | **Location where item is reported** |
| --- | --- | --- | --- |
| **TITLE** | | |  |
| Title | 1 | Identify the report as a systematic review. | Page 1 |
| **ABSTRACT** | | |  |
| Abstract | 2 | See the PRISMA 2020 for Abstracts checklist. | Page 2 |
| **INTRODUCTION** | | |  |
| Rationale | 3 | Describe the rationale for the review in the context of existing knowledge. | Page 4 |
| Objectives | 4 | Provide an explicit statement of the objective(s) or question(s) the review addresses. | Page 4 |
| **METHODS** | | |  |
| Eligibility criteria | 5 | Specify the inclusion and exclusion criteria for the review and how studies were grouped for the syntheses. | Page 6 |
| Information sources | 6 | Specify all databases, registers, websites, organisations, reference lists and other sources searched or consulted to identify studies. Specify the date when each source was last searched or consulted. | Page 5 |
| Search strategy | 7 | Present the full search strategies for all databases, registers and websites, including any filters and limits used. | Page 5 |
| Selection process | 8 | Specify the methods used to decide whether a study met the inclusion criteria of the review, including how many reviewers screened each record and each report retrieved, whether they worked independently, and if applicable, details of automation tools used in the process. | Page 7 |
| Data collection process | 9 | Specify the methods used to collect data from reports, including how many reviewers collected data from each report, whether they worked independently, any processes for obtaining or confirming data from study investigators, and if applicable, details of automation tools used in the process. | Pages 7 & 8 |
| Data items | 10a | List and define all outcomes for which data were sought. Specify whether all results that were compatible with each outcome domain in each study were sought (e.g. for all measures, time points, analyses), and if not, the methods used to decide which results to collect. | Pages 7 & 8 |
|  | 10b | List and define all other variables for which data were sought (e.g. participant and intervention characteristics, funding sources). Describe any assumptions made about any missing or unclear information. | Pages 7 & 8 |
| Study risk of bias assessment | 11 | Specify the methods used to assess risk of bias in the included studies, including details of the tool(s) used, how many reviewers assessed each study and whether they worked independently, and if applicable, details of automation tools used in the process. | Page 7 |
| Effect measures | 12 | Specify for each outcome the effect measure(s) (e.g. risk ratio, mean difference) used in the synthesis or presentation of results. | Pages 8 & 9 |
| Synthesis methods | 13a | Describe the processes used to decide which studies were eligible for each synthesis (e.g. tabulating the study intervention characteristics and comparing against the planned groups for each synthesis (item #5)). | Page 8 |
|  | 13b | Describe any methods required to prepare the data for presentation or synthesis, such as handling of missing summary statistics, or data conversions. | Pages 8 & 9 |
|  | 13c | Describe any methods used to tabulate or visually display results of individual studies and syntheses. | n/a |
|  | 13d | Describe any methods used to synthesize results and provide a rationale for the choice(s). If meta-analysis was performed, describe the model(s), method(s) to identify the presence and extent of statistical heterogeneity, and software package(s) used. | Pages 8 & 9 |
|  | 13e | Describe any methods used to explore possible causes of heterogeneity among study results (e.g. subgroup analysis, meta-regression). | Page 9 |
|  | 13f | Describe any sensitivity analyses conducted to assess robustness of the synthesized results. | Page 9 |
| Reporting bias assessment | 14 | Describe any methods used to assess risk of bias due to missing results in a synthesis (arising from reporting biases). | Page 9 |
| Certainty assessment | 15 | Describe any methods used to assess certainty (or confidence) in the body of evidence for an outcome. | Page 9 |
| **RESULTS** | | |  |
| Study selection | 16a | Describe the results of the search and selection process, from the number of records identified in the search to the number of studies included in the review, ideally using a flow diagram. | Page 10, Figure 1 |
|  | 16b | Cite studies that might appear to meet the inclusion criteria, but which were excluded, and explain why they were excluded. | n/a |
| Study characteristics | 17 | Cite each included study and present its characteristics. | Page 10, Table 1, Appendices B & C |
| Risk of bias in studies | 18 | Present assessments of risk of bias for each included study. | Page 11, Appendix D |
| Results of individual studies | 19 | For all outcomes, present, for each study: (a) summary statistics for each group (where appropriate) and (b) an effect estimate and its precision (e.g. confidence/credible interval), ideally using structured tables or plots. | Figures 2-5 |
| Results of syntheses | 20a | For each synthesis, briefly summarise the characteristics and risk of bias among contributing studies. | Pages 11-15 |
|  | 20b | Present results of all statistical syntheses conducted. If meta-analysis was done, present for each the summary estimate and its precision (e.g. confidence/credible interval) and measures of statistical heterogeneity. If comparing groups, describe the direction of the effect. | Pages 11-15, Figures 2-5 |
|  | 20c | Present results of all investigations of possible causes of heterogeneity among study results. | Pages 13 & 14, Appendices E-K |
|  | 20d | Present results of all sensitivity analyses conducted to assess the robustness of the synthesized results. | Pages 13 & 14, Appendices E-K |
| Reporting biases | 21 | Present assessments of risk of bias due to missing results (arising from reporting biases) for each synthesis assessed. | Pages 13 & 14, Appendices E-K |
| Certainty of evidence | 22 | Present assessments of certainty (or confidence) in the body of evidence for each outcome assessed. | Pages 13 & 14, Appendices E-K |
| **DISCUSSION** | | |  |
| Discussion | 23a | Provide a general interpretation of the results in the context of other evidence. | Pages 16 & 17 |
|  | 23b | Discuss any limitations of the evidence included in the review. | Page 18 |
|  | 23c | Discuss any limitations of the review processes used. | Page 18 |
|  | 23d | Discuss implications of the results for practice, policy, and future research. | Page 18 |
| **OTHER INFORMATION** | | |  |
| Registration and protocol | 24a | Provide registration information for the review, including register name and registration number, or state that the review was not registered. | Page 5 |
|  | 24b | Indicate where the review protocol can be accessed, or state that a protocol was not prepared. | Page 5 |
|  | 24c | Describe and explain any amendments to information provided at registration or in the protocol. | n/a |
| Support | 25 | Describe sources of financial or non-financial support for the review, and the role of the funders or sponsors in the review. | n/a |
| Competing interests | 26 | Declare any competing interests of review authors. | Page 19 |
| Availability of data, code and other materials | 27 | Report which of the following are publicly available and where they can be found: template data collection forms; data extracted from included studies; data used for all analyses; analytic code; any other materials used in the review. | Page 19 |

**Supplementary Appendix B:** Demographic information of included studies (i).

| Source | Study Methodology | Sample | Sample Size | Age, y | Cases Age | Controls Age | Baseline PANSS Total (Cases) | Baseline PANSS Total (Controls) |
| --- | --- | --- | --- | --- | --- | --- | --- | --- |
| *Suicide*  Reutfors et al, 2009 (Sweden)  Dutta et al, 2011 (UK)  Koola et al, 2012 (USA)  Hjorthoj et al, 2015 (Denmark)  Ostergaard et al, 2017 (Denmark)  Lahteenvuo et al, 2021 (Finland)  Lahteenvuo et al, 2021 (Sweden)  Bornheimer et al, 2024 (USA)  *Attempted Suicide*  Dervaux et al, 2003 (France)  Robinson et al, 2009 (Australia)  Makkos et al, 2011 (Hungary)  McLean et al, 2012 (Australia)  Mauri et al, 2013 (Italy)  Luckoff et al, 2014 (South Africa) – U, A & D  Ayesa-Arriola et al, 2015 (Spain)  Adan et al, 2017 (Spain) – Males  Ostergaard et al, 2017 (Denmark)  Naji et al, 2018 (Canada) – Males  Naji et al, 2018 (Canada) - Females  Waterreus et al, 2018 (Germany) – Males  Waterreus et al, 2018 (Germany) – Females  Lopez-Morinigo et al, 2019 (UK) – GAP  Toll et al, 2023 (Spain)  Fekih-Romdhane et al, 2023 (Tunisia)  Fridman et al, 2023 (Israel)  Golay et al, 2023 (Switzerland)  Koubaa et al, 2023 (Morocco)  Sastre-Buades et al, 2023 (Spain)  Phalen et al, 2024 (USA)  Ricci et al, 2024 (Italy)  *Suicidal Ideation*  Salagre et al, 2020 (Spain)  Fridman et al, 2023 (Israel)  Sicotte et al, 2023 (Canada)  Heuschen et al, 2024 (The Netherlands)  Phalen et al, 2024 (USA)  Ricci et al, 2024 (Italy) | Cross-Sectional / Case Control  Longitudinal  Longitudinal  Longitudinal  Longitudinal  Longitudinal  Longitudinal  Cross-Sectional / Case Control  Cross-Sectional / Case Control  Cross-Sectional / Case Control  Cross-Sectional / Case Control  Cross-Sectional / Case Control  Cross-Sectional / Case Control  Cross-Sectional / Case Control  Longitudinal  Cross-Sectional / Case Control  Longitudinal  Cross-Sectional / Case Control  Cross-Sectional / Case Control  Cross-Sectional / Case Control  Cross-Sectional / Case Control  Cross-Sectional / Case Control  Cross-Sectional / Case Control  Cross-Sectional / Case Control  Cross-Sectional / Case Control  Longitudinal  Cross-Sectional / Case Control  Cross-Sectional / Case Control  Longitudinal  Longitudinal  Longitudinal  Cross-Sectional / Case Control  Longitudinal  Cross-Sectional / Case Control  Longitudinal  Longitudinal | Chronic  FEP  Chronic  Chronic  Chronic  Chronic  Chronic  Chronic  Chronic  FEP  Chronic  Chronic  Chronic  Chronic  FEP  Chronic  Chronic  Chronic  Chronic  Chronic  Chronic  FEP  FEP  FEP  Chronic  FEP  Chronic  FEP  FEP  FEP  FEP  Chronic  FEP  FEP  FEP  FEP | 168  1575  762  41,470  35,625  30,860  14,616  57  34  658  85  812  106  980  397  50  35,625  364  314  1065  725  112  267  33  144  269  304  190  1101  42  302  144  352  551  1101  43 | -  -  37.3 (8.8)  27.6 (9.2)  27.6 (8.8)  -  -  45.8 (15.7)  -  -  -  -  48.3 (12.7)  -  28.9 (9.5)  36.1 (7.8)  -  -  -  -  -  29.4 (9.2)  -  -  -  24.3 (-)  -  27.9 (7.4)  -  -  -  -  -  32.4 (10.9)  -  - | 32.8 (9.7)  -  -  -  -  -  -  42.0 (18.7)  28.9 (6.3)  21.5 (3.6)  24.5 (4.4)  -  47.4 (14.0)  -  28.4 (8.6)  36.2 (7.0)  -  -  -  -  -  27.8 (5.8)  24.0 (8.5)  -  36.6 (10.3)  -  35.5 (9.2)  27.4 (8.0)  -  24.6 (5.2)  26.2 (11.0)  36.6 (10.3)  22.3 (2.95)  -  -  24.6 (5.2) | 32.8 (9.7)  -  -  -  -  -  -  49.2 (11.7)  37.0 (12.7)  22.1 (3.4)  31.3 (3.0)  -  48.7 (12.0)  -  30.1 (9.6)  35.9 (8.3)  -  -  -  -  -  29.8 (5.8)  25.3 (8.1)  -  39.5 (10.5)  -  37.9 (11.3)  28.0 (7.3)  -  25.1 (4.3)  23.2 (9.0)  39.5 (10.5)  23.6 (2.7)  -  -  25.1 (4.3) | -  -  -  -  -  -  -  -  76.2 (11.0)  -  -  -  -  -  -  -  -  -  -  -  -  -  89.7 (22.0)  -  75.0 (33.0)  -  -  57.1 (16.5)  -  -  75.0 (33.0)  -  -  -  -  - | -  -  -  -  -  -  -  -  79.7 (14.4)  -  -  -  -  -  -  -  -  -  -  -  -  -  82.2 (21.6)  -  77.0 (23.0)  -  -  58.7 (18.1)  -  -  77.0 (23.0)  -  -  -  -  - |

Note: A, Abuse; D, Dependence; FEP, First Episode Psychosis; GAP, Genetics and Psychosis; PANSS, Positive and Negative Syndrome Scale; U, Use.

**Supplementary Appendix C:** Demographic information of included studies (ii).

| Source | Diagnosis | Diagnosis Measure | Drugs Measure | Drugs Timeframe | Suicidality Measure |
| --- | --- | --- | --- | --- | --- |
| *Suicide*  Reutfors et al, 2009 (Sweden)  Dutta et al, 2011 (UK)  Koola et al, 2012 (USA)  Hjorthoj et al, 2015 (Denmark)  Ostergaard et al, 2017 (Denmark)  Lahteenvuo et al, 2021 (Finland)  Lahteenvuo et al, 2021 (Sweden)  Bornheimer et al, 2024 (USA)  *Attempted Suicide*  Dervaux et al, 2003 (France)  Robinson et al, 2009 (Australia)  Makkos et al, 2011 (Hungary)  McLean et al, 2012 (Australia)  Mauri et al, 2013 (Italy)  Luckoff et al, 2014 (South Africa) – U, A & D  Ayesa-Arriola et al, 2015 (Spain)  Adan et al, 2017 (Spain) – Males  Ostergaard et al, 2017 (Denmark)  Naji et al, 2018 (Canada) – Males  Naji et al, 2018 (Canada) – Females  Waterreus et al, 2018 (Germany) – Males  Waterreus et al, 2018 (Germany) – Females  Lopez-Morinigo et al, 2019 (UK) – GAP  Toll et al, 2023 (Spain)  Fekih-Romdhane et al, 2023 (Tunisia)  Fridman et al, 2023 (Israel)  Golay et al, 2023 (Switzerland)  Koubaa et al, 2023 (Morocco)  Sastre-Buades et al, 2023 (Spain)  Phalen et al, 2024 (USA)  Ricci et al, 2024 (Italy)  *Suicidal Ideation*  Salagre et al, 2020 (Spain)  Fridman et al, 2023 (Israel)  Sicotte et al, 2023 (Canada)  Heuschen et al, 2024 (The Netherlands)  Phalen et al, 2024 (USA)  Ricci et al, 2024 (Italy) | F20, F25, 295  SCZ, SCA, BD, PDep, SCP  SCZ, SCA, PDNOS  SCZ  F20-25, F28-29, 295, 297, 298, 299, 301  F20, F25, 295  F20, F25  SCZ, SCA, DepD, PDep  SCZ, SCA  SCZ, SCA, SCP, DD, PDNOS BD-I, BD-II, MDD  PSY  SCZ, SCA  SCZ  SCZ, SCA  SCZ, SCA, SCP, BPD  DS  F20-25, F28-29, 295, 297, 298, 299, 301  SCZ, SCP, SCA, DD, BPD  SCZ, SCP, SCA, DD, BPD  PD  PD  F10-F29, F30-F33  SCZ, BPD, SPD, UP, BD-I, SCA  FEP  SCZ  FEP  SCZ  FEP  FEP  FEP  FEP  SCZ  SSD  FEP  FEP  FEP | ICD-10, ICD-9, ICD-8  OPCRIT  Medical Records  ICD-10, ICD-8  ICD-10, ICD-8  ICD-10, ICD-9, ICD-8  ICD-10  DSM-IV  DSM-III-R  DSM-III-R, DSM-IV  ICD-10  DIGS, DSM-IV  DSM-IV-TR  DSM-IV-TR  DSM-IV  DSM-IV-TR  ICD-10, ICD-8  MINI  MINI  Psychosis Screener / Diagnostic Interview  Psychosis Screener / Diagnostic Interview  ICD-10  DSM-IV-TR  CAARMS  ICD-10  CAARMS  DSM--V  DSM-IV-TR  DSM-V  DSM-V  DSM-IV  ICD-10  DSM-IV-TR  ICD-10  DSM-V  DSM-V | Medical Records  OPCRIT, Medical Records  Medical Records, Urine Toxicology  ICD-10, ICD-8, ATC Codes  ICD-8, ICD-10, ATC Codes  ICD-9, ICD-10  ICD-9, ICD-10  Medical Records  CIDI  Early Psychosis File Questionnaire  ICD-10, Urine Analysis  DIGS  SCID-I  DIGS  Study Questionnaire  SCID-1, Research Interview  ICD-9, ICD-10  MINI  MINI  Research Interview  Research Interview  Study Questionnaire  Research Interview, SCID-I  Medical Records  Medical Records  DSM-IV  Study Questionnaire, Medical Records  Medical Records (Corroborated)  Research Interview  CUDIT-R  SCID-I, SCID-II  Medical Records  DUS  CEQ_EU-GEI_  Research Interview  CUDIT-R | Lifetime  Lifetime  Lifetime  Lifetime  Current, Former  Lifetime  Lifetime  Lifetime, < 3 months  Lifetime  Lifetime  Lifetime  Lifetime  Lifetime  Lifetime  Lifetime  Lifetime  Current, Former  < 12 months  < 12 months  < 12 months  < 12 months  Current  Lifetime  Lifetime  Lifetime  Lifetime  Lifetime  Lifetime  < 6 months, 1 month  < 6 months  Lifetime  Lifetime  Lifetime  Current, Lifetime  < 1 month  < 6 months | Cause of Death Register  Death Certificates, ICD-7, ICD-8, ICD-9, 1CD-10  Social Security Death Index  Cause of Death Register  Cause of Death Register  Medical Records, Death Register  Medical Records, Death Register  Medical Records, Legal Records  Medical Records  ICD-10  Medical Records  DIGS (Corroborated)  Research Interview (Corroborated)  DIGS (Corroborated)  Medical Records  SCID-I, Medical Records  National Patient Register, ICD-10, ICD-8  MINI  MINI  NSMHW  NSMHW  Medical Records  Research Interview (Corroborated)  Medical Records  Medical Records  Medical Records  Study Questionnaire, Medical Records  Medical Records (Corroborated)  Clinician Rated  Medical Records  MADRS  Medical Records  Research Interview, Chart Review  CAPE  Clinician Rated, CSI  SSI |

Note: A, Abuse; ATC, Anatomic Therapeutic Chemical; BD-I, Bipolar Disorder I; BD-II, Bipolar Disorder II; BPD, Brief Psychotic Disorder; CAARMS, Comprehensive Assessment of At Risk Mental States; CAPE, Community Assessment of Psychic Experience; CSI, Colorado Symptom Index; C-SSRS, Columbia-Suicide Severity Rating Scale; CUDIT-R, Cannabis Use Disorder Identification Test – Revised; D, Dependence; DD, Delusional Disorder; DepD, Depressive Disorder; DIGS, Diagnostic Interview for Genetic Studies; DS, Dual Schizophrenia; DSM, Diagnostic and Statistical Manual; DUS, Drug Use Scale; FEP; First Episode Psychosis; GAP, Genetics and Psychosis; ICD, International Classification for Diseases; MDD, Major Depressive Disorder; MADRS, Montgomery-Asberg Depression Rating Scale; MINI, Mini-International Neuropsychiatric Interview; NSMHW, National Survey of Mental Health and Wellbeing; OPCRIT, Operational Criteria Checklist for Psychotic Illness and Affective Illness; PD, Psychotic Disorder; PDep, Psychotic Depression; PDNOS, Psychotic Disorder Not Otherwise Specified; PSY, Psychosis; SCA; Schizoaffective Disorder; SCID, Structured Clinical Interview for DSM; SCP, Schizophreniform Disorder; SCZ, Schizophrenia; SSD, Schizophrenia Spectrum Disorder; SSI, Scale for Suicide Ideation; U, Use; UP, Unspecified Psychosis.

**Supplementary Appendix D**: Risk of Bias Assessment – Cannabis / Suicide-Related Outcomes

| Study | Selection Bias | Confounders | Data Collection | Dropouts | Analysis | Global |
| --- | --- | --- | --- | --- | --- | --- |
| *Suicide*  Reutfors et al, 2009  Dutta et al, 2011  Koola et al, 2012  Hjorthoj et al, 2015  Ostergaard et al, 2017  Lahteenvuo et al, 2021  Bornheimer et al, 2024 | 2  1  2  1  1  1  2 | 1  1  1  3  3  3  3 | 1  2  2  1  1  1  2 | 1  1  1  1  1  1  1 | 1  1  1  1  1  1  1 | 1  1  1  2  2  2  2 |
| *Attempted Suicide*  Dervaux et al, 2003  Robinson et al, 2009  Makkos et al, 2011  McLean et al, 2012  Mauri et al, 2013  Luckoff et al, 2014  Ayesa-Arriola et al, 2015  Adan et al, 2017  Ostergaard et al, 2017  Naji et al, 2018  Waterreus et al, 2018  Lopez-Morinigo et al, 2019  Toll et al, 2023  Fekih-Romdhane et al, 2023  Fridman et al, 2023  Golay et al, 2023  Koubaa et al, 2023  Sastre-Buades et al, 2023  Phalen et al, 2024  Ricci et al, 2024  *Suicidal Ideation*  Salagre et al, 2020  Fridman et al, 2023  Sicotte et al, 2023  Heuschen et al, 2024  Phalen et al, 2024  Ricci et al, 2024 | 2  1  2  1  2  3  2  3  1  3  1  2  1  1  2  2  3  2  1  2  3  2  3  1  1  2 | 3  1  2  2  2  1  3  2  3  2  1  3  1  1  3  2  3  1  1  3  2  3  2  2  1  3 | 1  1  1  1  1  2  1  1  2  1  1  1  1  2  1  1  1  1  1  1  1  1  1  1  1  1 | 1  1  1  1  1  1  1  2  1  1  1  2  1  2  1  1  1  2  2  1  2  1  2  2  2  1 | 2  1  3  1  1  2  1  1  1  2  1  1  1  3  1  1  1  2  1  2  1  1  1  1  1  2 | 2  1  2  1  1  2  2  2  2  2  1  2  1  2  2  1  3  2  1  2  2  2  2  1  1  2 |

Note: 1; Strong, 2; Moderate, 3; Weak

**Supplementary Appendix E**: Publication Bias - Alcohol / Attempted Suicide (k = 21) (ORs)

Odds Ratio - Egger’s Test of H0: No selection Bias (B = .22, SE = .13, *p* = 0.55).

**Supplementary Appendix F**: Sensitivity Analysis - Alcohol / Attempted Suicide (ORs)

| **Alcohol / Attempted Suicide - ORs** |  | |
| --- | --- | --- |
|  | Estimate if study removed | 95% Confidence Interval |
| Dervaux et al, 2003 | 1.34 | 1.14 – 1.57 |
| Robinson et al, 2009 | 1.42 | 1.17 – 1.73 |
| Makkos et al, 2011 | 1.43 | 1.20 – 1.71 |
| McLean et al, 2012 | 1.45 | 1.19 – 1.77 |
| Mauri et al, 2013 | 1.41 | 1.17 – 1.70 |
| Luckoff et al, 2014 | 1.43 | 1.16 – 1.77 |
| Ayesa-Arriola et al, 2015 | 1.38 | 1.14 – 1.67 |
| Adan et al, 2017 | 1.41 | 1.16 – 1.69 |
| Waterreus et al, 2018 - Males | 1.37 | 1.13 – 1.67 |
| Waterreus et al, 2018 - Females | 1.36 | 1.13 – 1.64 |
| Naji et al, 2018 - Males | 1.38 | 1.15 – 1.66 |
| Naji et al, 2018 - Females | 1.39 | 1.15 – 1.67 |
| Lopez-Morinigo et al, 2019 (GAP) | 1.39 | 1.15 – 1.68 |
| Fridman et al, 2023 | 1.41 | 1.18 – 1.70 |
| Golay et al, 2023 | 1.42 | 1.17 – 1.72 |
| Koubaa et al, 2023 | 1.32 | 1.12 – 1.56 |
| Toll et al, 2023 | 1.42 | 1.17 – 1.71 |
| Fekih-Romdhane et al, 2023 | 1.41 | 1.18 – 1.68 |
| Sastre-Buades et al, 2023 | 1.40 | 1.16 – 1.69 |
| Phalen et al, 2024 | 1.43 | 1.17 – 1.75 |
| Ricci et al, 2024 | 1.40 | 1.16 – 1.69 |

**Supplementary Appendix G**: Subgroup Analysis by Research Design – Cannabis / Attempted Suicide (ORs)


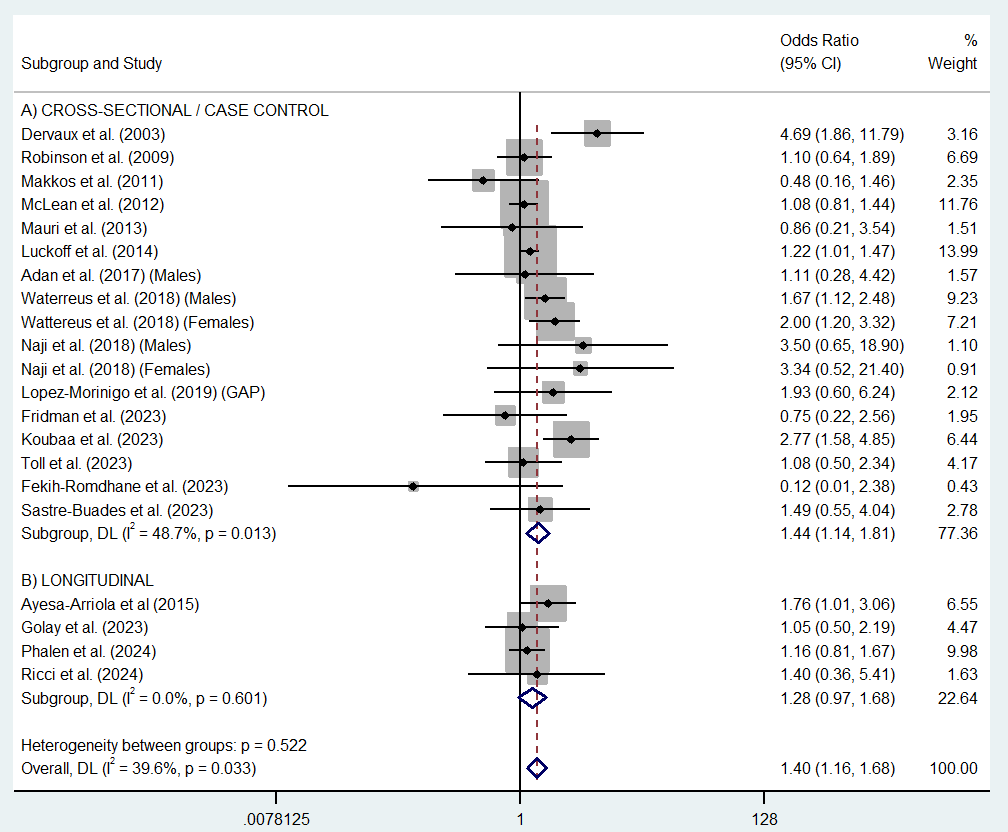


**Supplementary Appendix H**: Subgroup Analysis by Gender – Cannabis / Attempted Suicide (ORs)


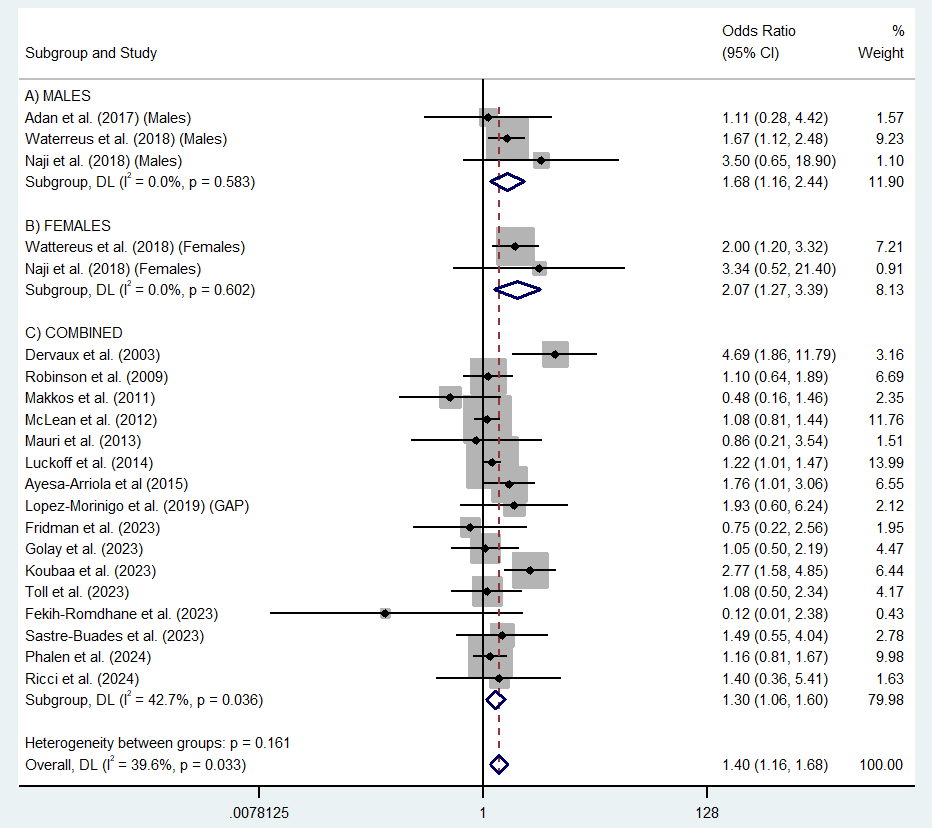


**Supplementary Appendix I**: Subgroup Analysis by Illness Course – Cannabis / Attempted Suicide (ORs)


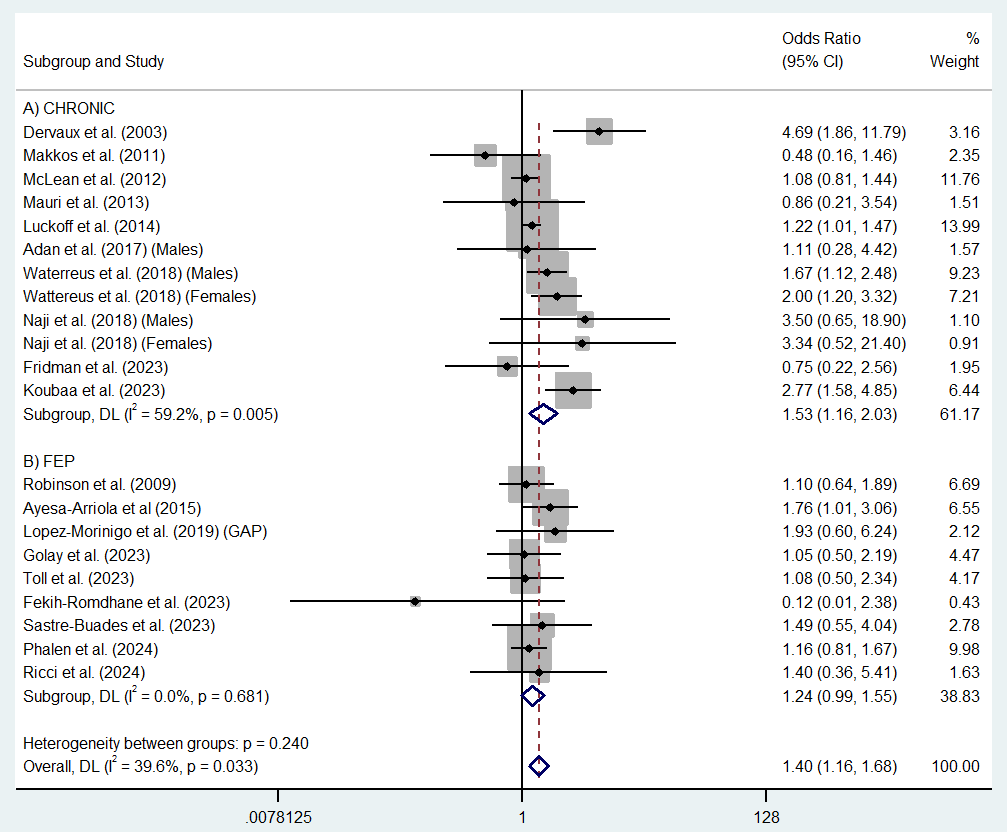


**Supplementary Appendix J**: Subgroup Analysis by Quality – Cannabis / Attempted Suicide (ORs)


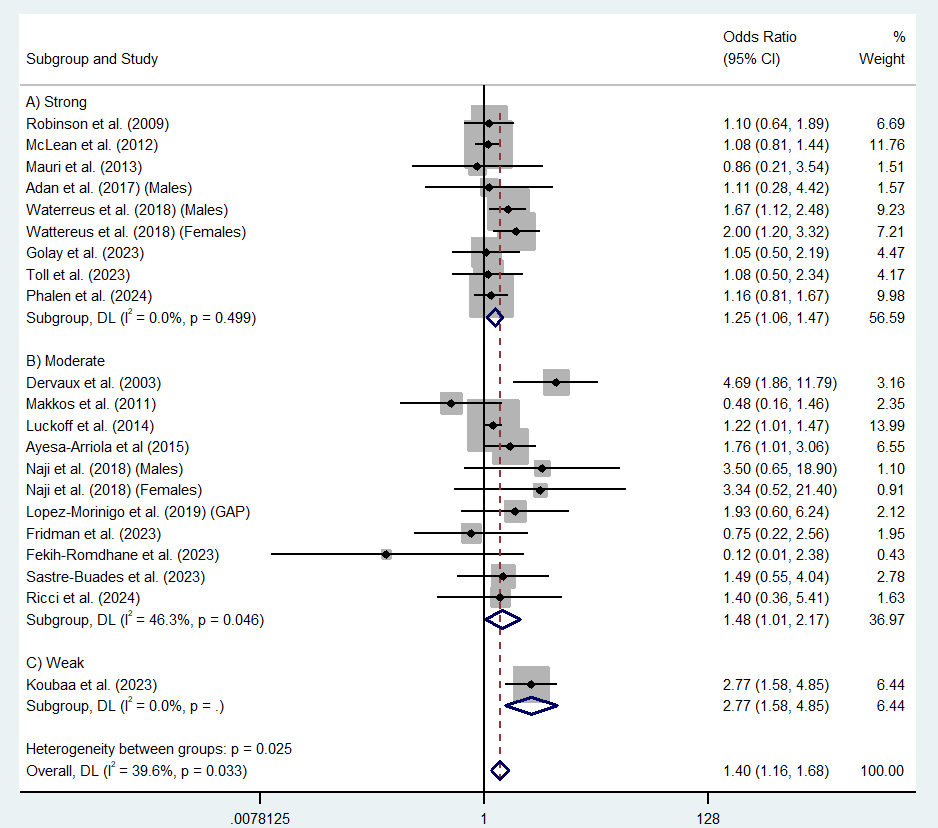


**Supplementary Appendix K**: Subgroup Analysis Table – Cannabis / Attempted Suicide (ORs)

| Variable | No. of studies | Pooled OR (95% CI) | Homogeneity within the study (I^2^ and Q) | |
| --- | --- | --- | --- | --- |
|  | | | Q value | I^2,^ *p*-value |
| *Study Design* |  |  |  |  |
| Cross-Sectional / Case Control | 17 | 1.44 (1.14 – 1.81) | 31.38 | 48.7% |
| Longitudinal | 4 | 1.28 (0.97 – 1.68) | 1.86 | 0.0% |
|  |  |  |  |  |
| *Gender* |  |  |  |  |
| Male | 3 | 1.68 (1.16 – 2.44) | 1.08 | 0.0% |
| Female | 2 | 2.07 (1.27 – 3.39) | 0.27 | 0.0% |
| Combined | 16 | 1.30 (1.06 – 1.60) | 26.16 | 42.7% |
|  |  |  |  |  |
| *Sample* |  |  |  |  |
| Chronic | 12 | 1.53 (1.16 – 2.03) | 26.97 | 59.2% |
| First Episode Psychosis (FEP) | 9 | 1.24 (0.99 – 1.55) | 5.70 | 0.0% |
|  |  |  |  |  |
| *Study Quality* |  |  |  |  |
| Weak | 1 | 2.77 (1.58 – 4.85) | 0 | - |
| Moderate | 11 | 1.48 (1.01 – 2.17) | 18.61 | 46.3% |
| Strong | 9 | 1.25 (1.06 – 1.47) | 7.35 | 0.0% |

Note: OR, Odds Ratio.

**Supplementary References**

Adan, A., Capella, M. D. M., Prat, G., Forero, D. A., López-Vera, S., & Navarro, J. F. (2017). Executive functioning in men with schizophrenia and substance use disorders. Influence of lifetime suicide attempts. *PloS One*, *12*(1), e0169943.

Ayesa-Arriola, R., Alcaraz, E. G., Hernández, B. V., Pérez-Iglesias, R., Moríñigo, J. D. L., Duta, R., ... & Crespo-Facorro, B. (2015). Suicidal behaviour in first-episode non-affective psychosis: Specific risk periods and stage-related factors. *European Neuropsychopharmacology*, *25*(12), 2278-2288.

Bornheimer, L. A., Bagge, C., Overholser, J., Brdar, N. M., Matta, N., Kitchen, M., ... & Stockmeier, C. A. (2024). Demographic and clinical characteristics of individuals with psychosis symptoms who died by suicide: Findings of a psychological autopsy study. *Psychiatry Research*, 116185.

Dervaux, A., Laqueille, X., Bourdel, M. -C., LeBorgne, M. -H., Olié, J. -P., Lóo, H., & Krebs, M. -O. (2003). Cannabis et schizophrénie: Données cliniques et socio-démographiques [Cannabis and schizophrenia: Demographic and clinical correlates]. *L'Encéphale: Revue de psychiatrie clinique biologique et thérapeutique, 29*(1), 11–17.

Dutta, R., Murray, R. M., Allardyce, J., Jones, P. B., & Boydell, J. (2011). Early risk factors for suicide in an epidemiological first episode psychosis cohort. *Schizophrenia Research*, *126*(1-3), 11-19.

Fekih-Romdhane, F., Abassi, B., Ghrissi, F., Loch, A. A., Cherif, W., Damak, R., ... & Cheour, M. (2023). Suicide risk among individuals at Ultra-High Risk (UHR) of psychosis in a developing North African country: a 12-month naturalistic prospective cohort study from the TRIP project. *Psychiatry Research*, *327*, 115409.

Fridman, J., Bloemhof-Bris, E., Weizman, S., Kessler, T., Porat, D., Ivry, A., ... & Shelef, A. (2023). Inflammation Markers Among Schizophrenia Patients Who Use Cannabis. *Clinical Neuropharmacology*, *46*(4), 145-148.

Golay, P., Reitzel, E., & Conus, P. (2023). The impact of established risk factors for psychosis on the 3-year outcomes. *Swiss Archives of Neurology, Psychiatry & Psychotherapy*, *174*(03), 88-92.

Heuschen, C. B. B. C. M., Bolhuis, K., Zantvoord, J. B., Bockting, C. L., Denys, D. A. J. P., Lok, A., ... & Schirmbeck, F. (2024). Self-reported suicidal ideation among individuals with first episode psychosis and healthy controls: Findings from the international multicentre EU-GEI study. *Schizophrenia Research*, *270*, 339-348.

Hjorthøj, C., Østergaard, M. L. D., Benros, M. E., Toftdahl, N. G., Erlangsen, A., Andersen, J. T., & Nordentoft, M. (2015). Association between alcohol and substance use disorders and all-cause and cause-specific mortality in schizophrenia, bipolar disorder, and unipolar depression: a nationwide, prospective, register-based study. *The Lancet Psychiatry*, *2*(9), 801-808.

Koola, M. M., McMahon, R. P., Wehring, H. J., Liu, F., Mackowick, K. M., Warren, K. R., ... & Kelly, D. L. (2012). Alcohol and cannabis use and mortality in people with schizophrenia and related psychotic disorders. *Journal of Psychiatric Research*, *46*(8), 987-993.

Koubaa, I., Aden, M. O., & Barrimi, M. (2023). Prevalence and factors associated with suicide attempts among Moroccan patients with schizophrenia: cross-sectional study. *Annals of Medicine & Surgery*, *85*(6), 2528-2533.

Lähteenvuo, M., Batalla, A., Luykx, J. J., Mittendorfer‐Rutz, E., Tanskanen, A., Tiihonen, J., & Taipale, H. (2021). Morbidity and mortality in schizophrenia with comorbid substance use disorders. *Acta Psychiatrica Scandinavica*, *144*(1), 42-49.

Lopez-Morinigo, J. D., Di Forti, M., Ajnakina, O., Wiffen, B. D., Morgan, K., Doody, G. A., ... & David, A. S. (2019). Insight and risk of suicidal behaviour in two first-episode psychosis cohorts: Effects of previous suicide attempts and depression. *Schizophrenia Research*, *204*, 80-89.

Lückhoff, M., Koen, L., Jordaan, E., & Niehaus, D. (2014). Attempted suicide in a Xhosa schizophrenia and schizoaffective disorder population. *Suicide & Life‐Threatening Behavior*, *44*(2), 167-174.

Makkos, Z., Fejes, L., Inczédy-Farkas, G., Kassai-Farkas, A., Faludi, G., & Lazary, J. (2011). Psychopharmacological comparison of schizophrenia spectrum disorder with and without cannabis dependency. *Progress in Neuro-Psychopharmacology & Biological Psychiatry*, *35*(1), 212-217.

Mauri, M. C., Paletta, S., Maffini, M., Moliterno, D., & Altamura, A. C. (2013). Suicide attempts in schizophrenic patients: clinical variables. *Asian Journal of Psychiatry*, *6*(5), 421-427.

McLean, D., Gladman, B., & Mowry, B. (2012). Significant relationship between lifetime alcohol use disorders and suicide attempts in an Australian schizophrenia sample. *Australian & New Zealand Journal of Psychiatry*, *46*(2), 132-140.

Naji, L., Rosic, T., Dennis, B., Bhatt, M., Sanger, N., Hudson, J., ... & Samaan, Z. (2018). The association between cannabis use and suicidal behavior in patients with psychiatric disorders: an analysis of sex differences. *Biology of Sex Differences*, *9*, 1-8.

Østergaard, M. L., Nordentoft, M., & Hjorthøj, C. (2017). Associations between substance use disorders and suicide or suicide attempts in people with mental illness: a Danish nation‐wide, prospective, register‐based study of patients diagnosed with schizophrenia, bipolar disorder, unipolar depression or personality disorder. *Addiction*, *112*(7), 1250-1259.

Phalen, P., Jones, N., Davis, B., Sarpal, D., Dickerson, F., Vatza, C., ... & Bennett, M. (2024). Suicidality among clients in a network of coordinated specialty care (CSC) programs for first-episode psychosis: Rates, changes in rates, and their predictors. *Schizophrenia Research*, *274*, 150-157.

Reutfors, J., Brandt, L., Jönsson, E. G., Ekbom, A., Sparén, P., & Ösby, U. (2009). Risk factors for suicide in schizophrenia: findings from a Swedish population-based case-control study. *Schizophrenia Research*, *108*(1-3), 231-237.

Ricci, V., Di Muzio, I., Ceci, F., Di Carlo, F., Mancusi, G., Piro, T., ... & Maina, G. (2024). Aberrant salience in cannabis-induced psychosis: a comparative study. *Frontiers in Psychiatry*, *14*, 1343884.

Robinson, J., Cotton, S., Conus, P., Graf Schimmelmann, B., McGorry, P., & Lambert, M. (2009). Prevalence and predictors of suicide attempt in an incidence cohort of 661 young people with first-episode psychosis. *Australian & New Zealand Journal of Psychiatry*, *43*(2), 149-157.

Salagre, E., Grande, I., Jiménez, E., Mezquida, G., Cuesta, M. J., Llorente, C., ... & PEPs Group. (2021). Trajectories of suicidal ideation after first‐episode psychosis: A growth mixture modeling approach. *Acta Psychiatrica Scandinavica*, *143*(5), 418-433.

Sastre-Buades, A., Caro-Cañizares, I., Ochoa, S., Lorente-Rovira, E., Barajas, A., Gutiérrez-Zotes, A., ... & Spanish Metacognition Study Group. (2023). Relationship between cognition and suicidal behavior in recent-onset psychosis. *Schizophrenia Research*, *252*, 172-180.

Sicotte, R., Iyer, S. N., Lacourse, É., Séguin, J. R., & Abdel-Baki, A. (2023). Heterogeneity in the course of suicidal ideation and its relation to suicide attempts in first-episode psychosis: a 5-year prospective study. *The Canadian Journal of Psychiatry*, *68*(11), 850-859.

Toll, A., Pechuan, E., Bergé, D., Legido, T., Martínez-Sadurní, L., El-Abidi, K., ... & Mané, A. (2023). Factors associated with suicide attempts in first-episode psychosis during the first two years after onset. *Psychiatry Research*, *325*, 115232.

Waterreus, A., Di Prinzio, P., Badcock, J. C., Martin-Iverson, M., Jablensky, A., & Morgan, V. A. (2018). Is cannabis a risk factor for suicide attempts in men and women with psychotic illness? *Psychopharmacology*, *235*, 2275-2285.
